# The potential of LLMs in generating questions and answers with EHRs

**DOI:** 10.3389/fdgth.2026.1799629

**Published:** 2026-07-15

**Authors:** Yunqi Zhu, Wen Tang, Huayu Yang, Jinghao Niu, Liyang Dou, Yifan Gu, Yuanyuan Wu, Wensheng Zhang, Ying Sun, Xuebing Yang

**Affiliations:** 1 Guangzhou University, Guangzhou, China; 2 Institute of Automation, Chinese Academy of Sciences, Beijing, China; 3 School of Information and Communication Engineering, Hainan University, Haikou, China; 4 Department of Geriatrics, Beijing Friendship Hospital, Capital Medical University, Beijing, China; 5 Xunfei Healthcare Technology Co., Ltd, Beijing, China

**Keywords:** artificial intelligence, electronic health record, large language model, medical exam, real-world application

## Abstract

**Background:**

This study aimed to generate medical qualification exam questions and their corresponding answers from real-world electronic health records (EHRs) with large language models (LLMs), and to compare their output to that of human medical experts.

**Methods:**

Utilizing a multicenter bidirectional anonymized database China Elderly Comorbidity Medical Database (CECMed), a total of 8 LLMs: ERNIE 4, ChatGLM 4, Doubao, Hunyuan, Spark 4, Qwen, Llama 3, and Mistral were tasked with generating open-ended questions and answers based on a subset of sampled admission reports. LLMs generated the medical question and answer through few-shot prompting. An independent expert panel scored the AI-generated outputs based on multiple criteria, including coherence, sufficiency of key information, information correctness, factual consistency, evidence of statement, and professionalism, using 5-point Likert scales.

**Results:**

For question generation, ERNIE 4 achieved the highest cumulative score (16.47). Human experts surpassed LLMs in sufficiency of key information (3.67) but lagged in information correctness (3.63 vs. LLMs' 4.03–4.57). The information correctness of ERNIE was significantly higher than the human's [0.93 (0.62, 1.24), *p* < 0.01]. For answer generation, humans led overall (14.49), while Doubao outperformed the other LLMs in coherence (3.57), factual consistency (3.60), and professionalism (3.53). The coherence of human's was significantly better than that of 8 LLMs, especially outperformed Llama [0.8 (0.37, 1.23), *p* < 0.01] and Mistral [0.87 (0.45, 1.28), *p* < 0.01].

**Conclusions:**

Conventional medical education requires clinicians to formulate questions and answers based on prototypes from EHRs, which is heuristic and time-consuming. This study shows that mainstream LLMs could generate questions and answers with real-world EHRs at levels close to clinicians. Although current LLMs performed dissatisfactorily in some aspects, medical students and interns may find LLMs a useful auxiliary tool to support their learning.

**Clinical Trial Registration:**

https://clinicaltrials.gov/study/NCT06316544, identifier: NCT06316544.

## 1 Introduction

Large language models (LLMs) have brought significant opportunities across various clinical applications. With the development of Transformer-based models (1–5), LLMs has great ability to process long contextual semantics for question-answering, enabling the generation of coherent and comprehensive response. Along this path, researchers have illuminated the convenience of LLMs and remarked some worrying aspects, such as the persistence of biases in the content generation within the medical domain (6), the pronounced reliance on demographic and disease-related decision-making in LLMs, the lack of interpretability and access to private medical data (7), etc. Suggestions have been made to employ data synthesis methods and interactive learning frameworks to elevate the quality of LLMs (8). In addition, the incorporation of multimodal information in LLMs is advocated to cater to the personalized needs of both patients and healthcare providers (9, 10).

Conventionally, AI models in medicine have primarily functioned as responders, tasked with simulating human-like responses in scenarios such as counselor in doctor-patient dialogues (11) and examinee in medical exams (12, 13). Existing studies, as synthesized in systematic reviews (14), have primarily focused on textbook-based multiple-choice questions with prompts (15–18), with limited exploration of open-ended question generation grounded in real-world electronic health records. Building on this prior work, our study investigates the feasibility of LLMs as exam question setters using clinical EHR data, addressing a gap in applied clinical education scenarios. Since LLMs are predominantly utilized as answer generators, whether LLMs can serve as a good examiner remains a critical challenge. Besides, real-world exams require the clinicians to write questions and answers by drawing upon prototypes from EHRs. This conventional paradigm is subjective, time-consuming, and limited in diversity. Therefore, this study explores the potential of a novel paradigm where LLMs act as question setters with EHRs to generate exam content. This exploratory attempt may offer a preliminary foundation for exploring personalized learning experiences for medical interns and residents, with the aim of informing ways to complement conventional medical education.

AI-Medicine integration faces challenges including interpretability and reliability and particularly the issue of hallucination (19–23), necessitate careful evaluation and mitigation strategies. Ensuring coherence, comprehensiveness, and professionalism is crucial for building trust in AI-generated content. Additionally, addressing biases, interpretability, and privacy concerns is vital for ethical deployment. In the context of medical education, specific challenges arise due to the nature of specialized medical knowledge and personalized experience. Furthermore, examples of human-authored subjective questions and answers are critical for transferring LLMs' knowledge to the target task. Meanwhile, there are no standardized benchmarks and evaluation metrics. Addressing these challenges requires a multidisciplinary approach, combining expertise in AI and medicine.

This absence of consensus on LLMs' evaluation metrics hinders the ability to compare the performance of different LLM. The evaluation has been approached with a set of criteria such as coherence, comprehensiveness, professionalism, and readability for diagnostic summary generation (24). Recently, the assessment of AI-generated medical advice has expanded to include empathy, comprehensiveness, and acceptance (25). Moreover, generative medical AI is expected to adhere to principles of accountability, model transparency, content privacy protection, fairness, and harmlessness (26). These principles are essential for ensuring the ethical and effective deployment of LLMs in healthcare, thereby overcoming the current limitations and realizing their full potential in transforming medical practice.

This work focuses on exploring whether LLMs can generate questions and answers for medical qualification exams using EHRs, which is an exploratory goal rather than conducting a comprehensive performance ranking of different LLMs. Unlike typical medical text generation tasks, such as doctor-patient dialogue, question-answering, report summarization etc. (27–31), which evaluate LLM's ability to simulate human responses, this task seeks to investigate whether models can contribute to educational contexts by creating exam. This work is grounded in a real-world Chinese dataset of elderly chronic diseases, utilizing few-shot prompting method for generating both the question and the answer, with the goal of simulating the question bank of AMBOSS Qbank (12) and USMLEasy (13). Specifically, we used a total of 8 LLMs: ERNIE 4.0–8K, ChatGLM-4-128K, Doubao-pro-32k, Hunyuan-turbo, Spark-4.0-Ultra, Qwen-2.5-max, Llama-3-70B-Instruct, and Mistral-7B (32–39), and the LLM-generated contents were accessed via their respective official commercial APIs. These LLMs are general-purpose models pre-trained on corpora containing both Chinese and English, though Llama and Mistral are more English-oriented. In addition, we also tasked LLMs with correcting and refining their original AI-generated answers by prompting a few samples of human experts' feedback on the AI-generated answers that have deficiencies.

**Figure 1:**
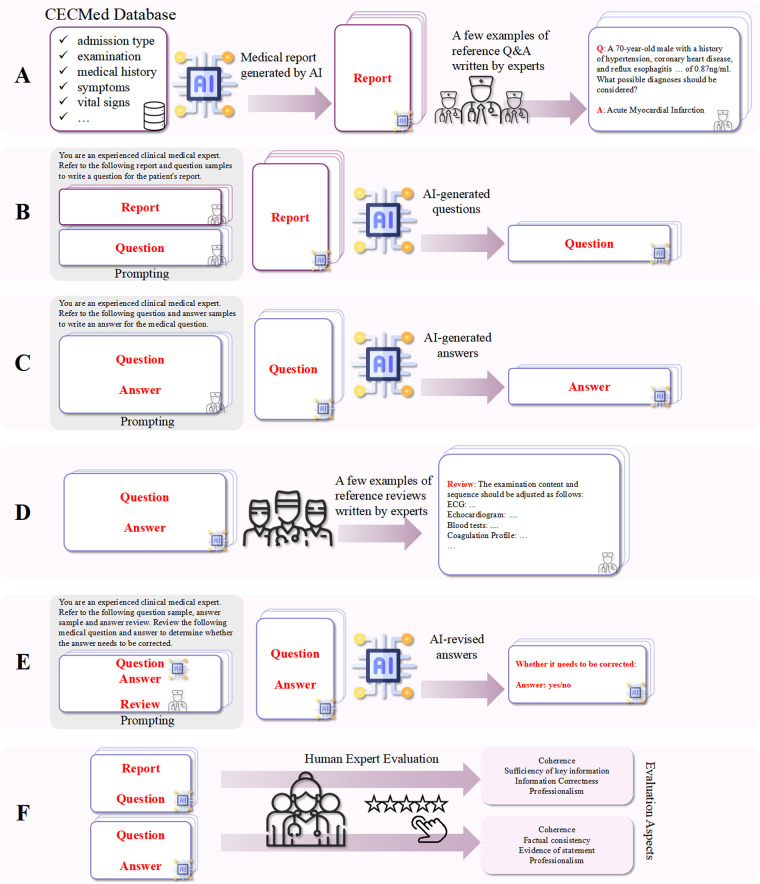
Overall pipeline.

For comparison, we engaged 3 doctors with 10 years of experience to author medical questions and answers based on the same reference cases and prompts. Human experts and the LLMs answered the same fixed set of human-validated questions to ensure comparability. For evaluation criteria, we refer to the aspects required for recent generative medical AI, such as comprehensiveness, consistency, readability, empathy, equity, non-maleficence, and accountability (24–26). Therefore, we have involved medical experts in the evaluation of the AI-generated content with a 5-point Likert Scale (40, 41) that a commonly employed scoring method for questionnaires. Specifically, based on established practices and research, our panel of medical and technical experts collaboratively selected the following evaluation criteria: we considered aspects of coherence, sufficiency of key information, information correctness, and professionalism as the evaluation criteria for question generation, and considered aspects of coherence, factual consistency, evidence of statement, and professionalism as the evaluation criteria for answer generation. The corresponding.explicit definitions of 5-point scoring and evaluation metrics are provided in Supplementary Material Table A1. Furthermore, 3 additional experienced doctors formed the evaluation panel. Figure 1 visualizes the overall pipeline of this study.

## 2 Methods

### 2.1 Data collection

We utilized a multicenter bidirectional anonymized database of older patients with comorbid chronic diseases [China Elderly Comorbidity Medical Database (CECMed)]. The retrospective cohort had enrollment from January 2010 to January 2022, while the prospective cohort had enrollment from January 2023 to November 2023. The patients were recruited from selected tertiary hospitals and community hospitals in southern, northern, and central regions of China. Inclusion criteria are as follows: (1) aged ≥ 65 years; (2) with at least one of the following typical five chronic diseases: coronary heart disease (CHD), hypertension, diabetes, chronic obstructive pulmonary disease (COPD) and osteoporosis. Exclusion criteria are as follows: (1) Late stage malignant tumors, expected survival time less than 3 months; (2) Completely disabled and unable to communicate; (3) Unable to cooperate with follow-up. As a result, the dataset is composed of a total of 2432 patient records. In the Supplementary Material Figure A1, we visualize the statistics of the original patient records.

For hospitalized patients, administrative information includes admission time, demographics, socioeconomic, past medical history, previous medication use, current symptoms, vital signs, laboratory tests and examinations, reasons for admission and admission diagnosis. All patients underwent comprehensive geriatric assessment within 24 h after admission. Barthel index, FRAIL, MORSE, MNA-SF and MINI-cog were used to assess patient's functional status. For outpatient patients, baseline data includes demographics, socioeconomic, medical history, medications, vital signs, comprehensive geriatric assessment and laboratory tests and examinations within the past three months. Patient data gathered was anonymized and preprocessed in a standardized manner. Furthermore, guided by semi-structured templates that integrate medical expert knowledge, we used ChatGLM to synthesize the information into admission reports and summaries.

### 2.2 Few-shot prompting

First, sampling patient reports from CECMed, human experts composed 4 reference questions and answers in an open-ended format. Subsequently, considering the time and cost constraints of a manageable human evaluation process, we determined a sample size of 30 (with effect size = 0.5, α err prob = 0.05, power = 0.5), and then randomly sampled 30 admission reports within the dataset and used the reference question as prompts to guide 8 different LLMs for the task of question generation. Next, under the guidance of medical experts panel, we selected the AI-generated questions, considering diversity and professionalism. We then randomly sampled 30 different questions, using the reference question-answer pairs as prompts, and instructed the LLMs to generate responses to these questions. In addition, taking into account both time and cost, medical experts provided a total of 20 concise open-ended reviews of the AI-generated answers, which included correct answers, incorrect answers and the reasons for errors. Further, we constructed prompts based on these answer reviews. As a result, we obtained 240 questions, 240 direct answer generations, and 43 AI-corrected answers. Noted that the reports and samples that involved human authorship and evaluation, as mentioned above, were not included in the final phase of human expert scoring.

Few-shot learning is an efficient strategy that leverages a small amount of annotated data to optimize machine learning models for domain-specific tasks. This study adopted the method of prompt engineering, enabling LLMs to emulate a limited number of reference questions and answers, thereby eliciting LLMs' knowledge in the medical specialties and adapting to this task. Noted that all LLMs were operated using their default generation settings, and the outputs were generated in a single run. Moreover, specific prompt engineering templates are available in the Supplementary Material Table A2. The original input texts are in Chinese. The original texts and their corresponding English translations (translated by ERNIE 4.0) are provided.

### 2.3 Human evaluation

We evaluated the performance of LLMs through human assessment. We involved a group of human experts (3 physicians with 10 years of clinical experience) to author 30 open-ended medical examination questions and answers based on the same reference cases and prompts (Supplementary Material Table A2). The EHR cases were randomly and evenly assigned to ensure independent work. All human-generated contents underwent the same pre-processing procedures applied to the LLM outputs. Importantly, to prevent potential bias, the human-authored contents were not used as reference materials for the LLMs' in-context learning. Further, the medical and technical experts collaboratively selected the evaluation criteria. Besides, the evaluation panel involved 3 independent experts, and they had clear understanding of the meaning of each evaluation criteria. The expert panel remained fully blinded to whether content was human- or LLM-authored, as well as to which specific model generated each item, throughout the entire scoring process. All the evaluators were clinical physicians from university-affiliated teaching hospitals who are also actively involved in medical education, including teaching undergraduate and postgraduate students and supervising residents. For the generation of questions, we assessed from four perspectives: coherence, sufficiency of key information, information correctness, and professionalism. Coherence refers to the contextual relevance and rationality between the viewpoints and sentences. Sufficient key information refers to the ability to extract and organize key information from the input, which is adequate for the examinee to formulate a reasonable response. Information correctness indicates whether the question is reliable, and whether the question contradicts factual knowledges. Professionalism is measured by the accuracy of terminology used, the relevance of medical knowledge content, adherence to professional ethical standards, and the depth of knowledge assessment; (2) For question-answering, the evaluation was based on the following four dimensions: coherence, factual consistency, evidence of statement, and professionalism. Coherence and professionalism were assessed identical to those mentioned above. Factual consistency evaluated whether LLMs' responses aligned with facts and addressed the question. The evidence of statement examined whether the reasoning and conclusions were supported by evidence within its response. Additionally, those AI-revised answers were randomly mixed with AI-generated answers in the evaluation phase. The scoring scales for evaluation metrics range from integers 1 to 5. For the statistical comparison, the formula for the mean is $\bar{x}=\frac{\sum_{i=1}^{n}\;x_{i}}{n}$, and the formula for the 95% confidence interval is $\bar{x}\pm Z\frac{\sigma }{\sqrt{n}}$.

## 3 Results

Table 1 details the mean scores and their corresponding 95% confidence interval of the evaluation results. For question generation, ERNIE 4 achieved the highest cumulative score (16.47). Qwen exhibited an overall inferior performance among 8 LLMs. Further, humans achieved a total of 15.33. We use the Friedman test to examine whether there are significant differences among different methods under the same evaluation criteria and consider the result of 8 LLMs to be significantly greater/smaller than human's when the *p*-value of the Wilcoxon signed-rank test is less than 0.05 (marked with *). Additionally, Bonferroni correction was adopted to address multiple comparisons across all evaluation dimensions, *p*-value derived from statistical tests remained statistically significant after the correction. It is noteworthy that no statistically significant differences were observed among the different methods in coherence, sufficiency of key information, and professionalism. Furthermore, humans scored higher than all AI models in terms of sufficiency of key information [3.67 (3.40, 3.93)] but lagged behind all LLMs in terms of information correctness [3.63 (3.37, 3.90)]. Moreover, information correctness of ERNIE is significantly higher than the human's [0.93 (0.62, 1.24), *p* < 0.01].

**Table 1:** Evaluation results. Question generation is assessed from four aspects: coherence (Coh.), sufficiency of key information (Suff. key info.), information correctness (Info. corr.), and professionalism (Prof.). Answer generation is assesssed from four aspects: coherence, factual consistency (Fact. cons.), evidence of statement (Evidence Stat.), and professionalism (Prof.). “Cumu”. stands for cumulative scores.

(a) Question generation					
Methods	Coh.	Suff. key info.	Info. corr.	Prof.	Cumu.
Human	4.13 [3.94, 4.32]	3.67 [3.40, 3.93]	3.63 [3.37, 3.90]	3.90 [3.70, 4.10]	15.33
ERNIE 4	4.30 [3.93, 4.67]	3.53 [3.16, 3.91]	4.57 [4.28, 4.86]*	4.07 [3.68, 4.46]	16.47
ChatGLM 4	4.17 [3.73, 4.61]	3.37 [2.93, 3.80]	4.43 [4.18, 4.69]*	3.70 [3.24, 4.16]	15.67
Doubao	4.13 [3.65, 4.62]	3.20 [2.71, 3.69]	4.03 [3.56, 4.51]	3.90 [3.46, 4.34]	15.26
Hunyuan	4.30 [3.87, 4.73]	3.33 [2.83, 3.84]	4.20 [3.73, 4.67]*	4.07 [3.56, 4.58]	15.90
Llama 3 70B	4.10 [3.68, 4.52]	3.47 [3.04, 3.89]	4.43 [4.06, 4.81]*	3.93 [3.47, 4.39]	15.93
Mistral 7B	4.43 [4.08, 4.78]	3.57 [3.18, 3.96]	4.43 [4.10, 4.77]*	3.90 [3.38, 4.42]	16.33
Qwen	3.83 [3.24, 4.43]	2.90 [2.43, 3.37]	4.07 [3.53, 4.60]	3.50 [2.92, 4.08]	14.30
Spark 4	3.80 [3.22, 4.38]	3.57 [3.09, 4.04]	4.23 [3.74, 4.73]*	3.70 [3.12, 4.28]	15.30

(b) Answer generation					
Methods	Coh.	Fact. Cons.	Evidence Stat.	Prof.	Cumu.
Human	3.93 [3.77, 4.10]	3.60 [3.39, 3.81]	3.53 [3.32, 3.75]	3.43 [3.18, 3.69]	14.49
ERNIE 4	3.47 [3.16, 3.77]*	3.43 [3.08, 3.78]	3.43 [3.11, 3.75]	3.37 [3.05, 3.68]	13.70
ChatGLM 4	3.23 [2.82, 3.65]*	3.33 [2.88, 3.79]	3.30 [2.84, 3.76]	3.20 [2.75, 3.65]	13.06
Doubao	3.57 [3.25, 3.89]*	3.60 [3.30, 3.90]	3.57 [3.23, 3.90]	3.53 [3.21, 3.85]	14.27
Hunyuan	3.33 [3.05, 3.62]*	3.50 [3.15, 3.85]	3.43 [3.08, 3.78]	3.27 [2.90, 3.63]	13.53
Llama 3 70B	3.13 [2.76, 3.51]*	3.10 [2.72, 3.48]	3.17 [2.80, 3.53]	3.03 [2.65, 3.42]	12.43
Mistral 7B	3.07 [2.70, 3.43]*	3.07 [2.69, 3.45]	3.07 [2.69, 3.45]	2.93 [2.58, 3.29]	12.14
Qwen	3.40 [3.04, 3.76]*	3.43 [3.04, 3.82]	3.40 [3.03, 3.77]	3.47 [3.08, 3.86]	13.70
Spark 4	3.53 [3.21, 3.85]*	3.57 [3.25, 3.89]	3.60 [3.28, 3.92]	3.33 [2.95, 3.72]	14.03

* We use the Friedman test to examine whether there are significant differences among different methods under the same evaluation criteria and consider the result of 8 LLMs to be significantly greater/smaller than human's when the *p*-value of the Wilcoxon signed-rank test is less than 0.05.

For answer generation, humans achieved the highest cumulative score across four evaluation metrics, totaling 14.49, while Doubao achieved the second, totaling 14.27. Mistral showed the overall least favorable performance among 8 LLMs. There is no statistically significant differences were observed among the different methods in the dimensions of factual consistency, evidence of statement, and professionalism. It was observed that humans achieved highest scores on coherence [3.93 (3.77, 4.10)] and factual consistency [3.60 (3.39, 3.81)]. Moreover, coherence of human's significantly outperformed that of 8 LLMs, particularly showing higher scores than the two English-based LLMs: Llama [0.8 (0.37, 1.23), *p* < 0.01] and Mistral [0.87 (0.45, 1.28), *p* < 0.01].

**Figure 2:**
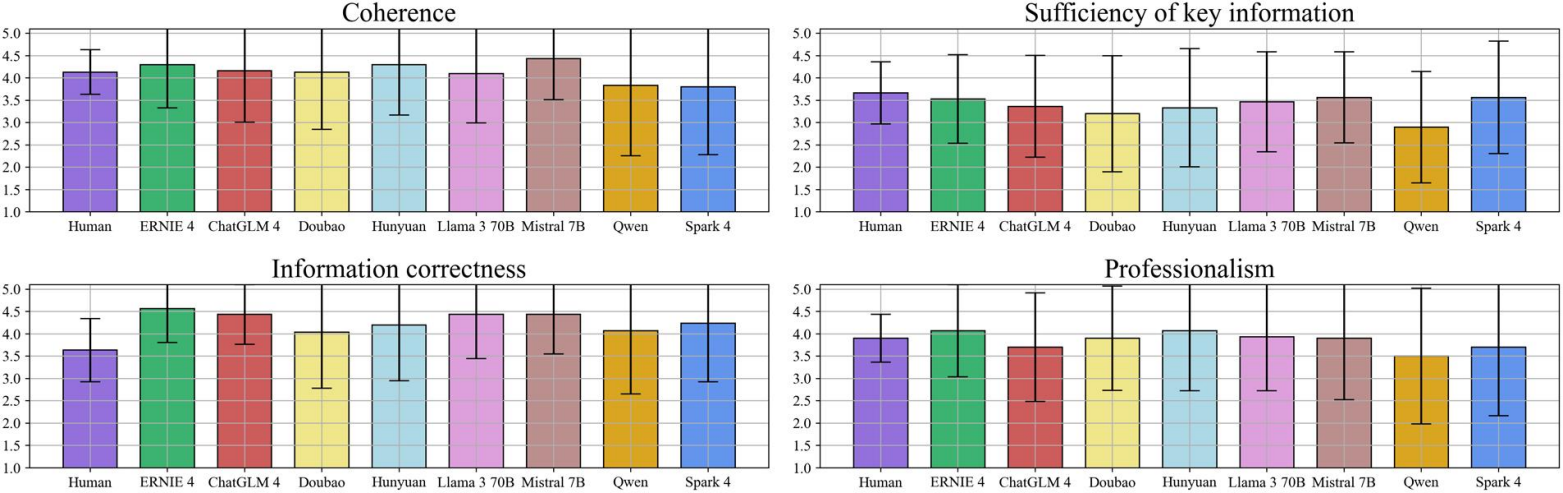
Human evaluation on question generation.

Figure 2 visualizes the results on question generation. The majority of LLMs achieve scores over 4 in terms of coherence and information correctness, and achieve scores nearly 4 in terms of professionalism, whereas the average score for sufficient of key information is relatively lower, with one scoring below 3.5 in general. This phenomenon may indicate the evaluators were satisfied with the correctness and readability of AI-generated questions. However, the LLMs still incur a loss of critical information during the process of extracting and abstracting information from the reports. Although the standard deviations of 8 LLMs were greater than the human's, they have achieved a similar criteria level.

**Figure 3:**
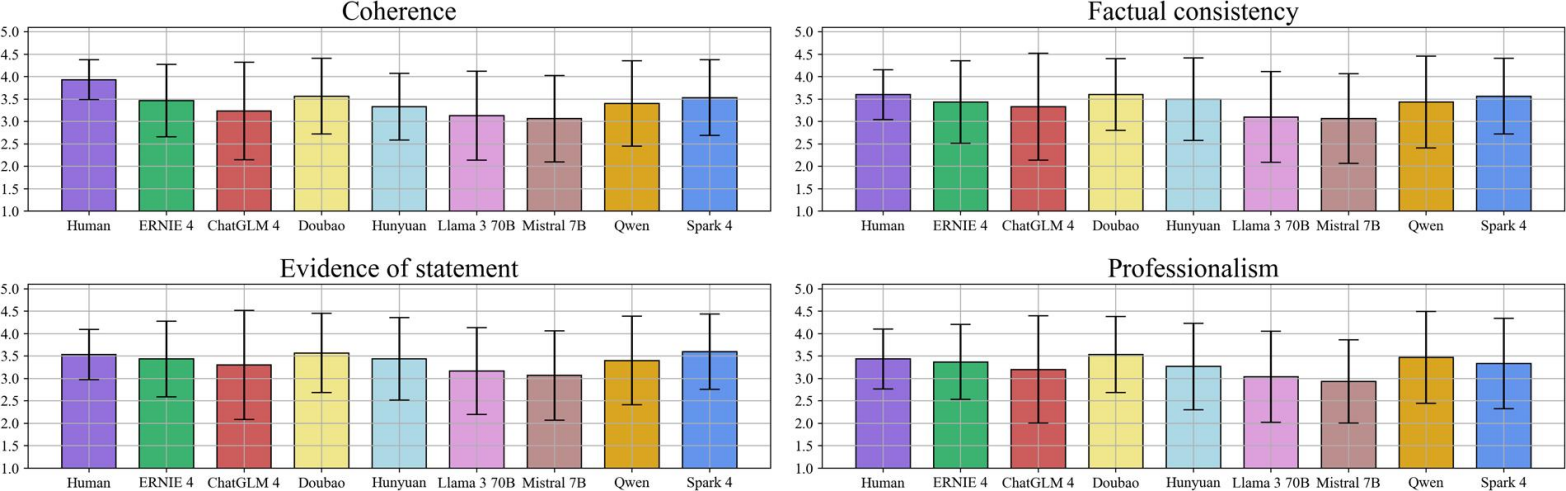
Human evaluation on answer generation.

Figure 3 visualizes the results on answer generation. It can be observed that the mean score for question-answering is generally lower than that for question generation, with LLMs' ratings hovering around 3.5 across all evaluation metrics. Additionally, the standard deviations of humans were generally smaller than LLMs', which indicates a significant gap between LLMs' responses and the expectation of human evaluators on medical open-ended question-answering.

**Figure 4:**
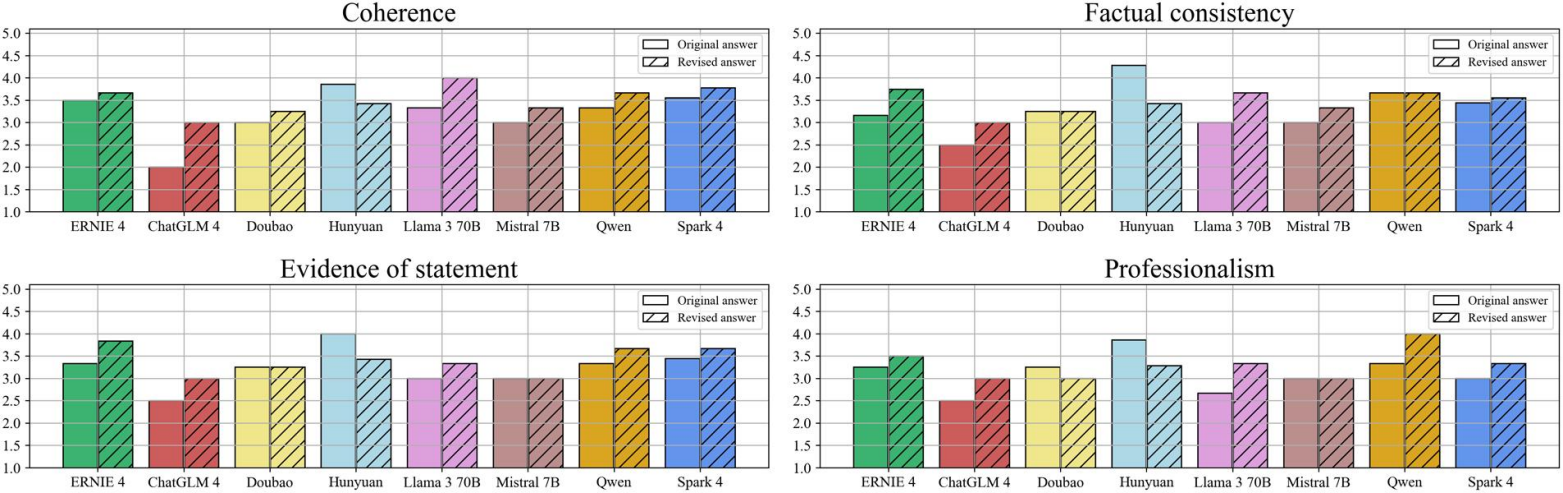
Human evaluation on answer revision.

Subsequently, we tasked medical experts with evaluating and correcting a subset of flawed AI-generated answers in the form of open-ended short text. We sampled these evaluations and corrections as new prompt engineering materials, allowing the model to judge whether a correction is needed for the sampled AI-generated question-answer pairs, and to provide new answers. We intermingled the answers revised by the model with those in the regular AI-generated answer in the evaluation phase. Figure 4 shows the comparison of directly generated answers and those modified by the LLMs. It can be observed that at least five models provide correction results that were more satisfactory, while the performance of the Hunyuan model slightly deteriorated. This unstable correction effects may be jointly driven by the model's domain adaptation capability in medical QA, and the limited number of correction samples.

In the Supplementary Material Figures A2(a),(b), we visualize the distributional relationship between the AI-generated questions and answers under different scores and the length of the generated text after the removal of stop words. In the Supplementary Material Figures A2(c),(d), we also visualize the distributional relationship between the proportion of words in the output text that do not appear in the input text under different scores. It can be observed that, except for a slight positive correlation between “sufficiency of key information” and text length, there is no obviously simple linear relationship between other evaluation metrics and text length or the proportion of novel words. The average text length for question generation and answer generation are 40.7, and 138.5, respectively. And then the proportion of novel words for question generation and answer generation are 7.4% and 68.8%, respectively.

Inter-rater reliability was evaluated using two-way mixed-effects intraclass correlation coefficients (ICC). We report both single-rater reliability ICC(2,1) and average-rater reliability ICC(2,3) for the three independent evaluators. For question generation, the overall ICC(2,1) was 0.596 and the overall ICC(2,3) was 0.810. Dimension-specific ICC values were: coherence [ICC(2,1) = 0.697, ICC(2,3) = 0.874], sufficiency of key information [ICC(2,1) = 0.446, ICC(2,3) = 0.707], information correctness [ICC(2,1) = 0.639, ICC(2,3) = 0.841], professionalism [ICC(2,1) = 0.603, ICC(2,3) = 0.819]. For answer generation, the overall ICC(2,1) was 0.141 and the overall ICC(2,3) was 0.328. Dimension-specific ICC values were: coherence [ICC(2,1) = 0.094, ICC(2,3) = 0.238], factual consistency [ICC(2,1) = 0.155, ICC(2,3) = 0.355], evidence of statement [ICC(2,1) = 0.158, ICC(2,3) = 0.359], and professionalism [ICC(2,1) = 0.159, ICC(2,3) = 0.362].

## 4 Discussion

This work explores LLMs' potential in generating medical exam pertaining to elderly chronic diseases through few-shot prompting, with the goal of simulating the question bank of AMBOSS Qbank (12) and USMLEasy (13). The observation that LLM can partially simulate real-world medical questions is encouraging, as it provides a preliminary reference for automated content generation in medicine. Nevertheless, a notable discrepancy in the quality of answers underscores the challenges of generating accurate, evidence-based, and professional responses. This emphasizes the necessity for further research and development to refine LLMs' comprehension and application of medical knowledge.

One potential direction is to explore the incorporation of medical knowledge base and expert feedback into LLMs using Retrieval-Augmented Generation. By integrating structured knowledge and leveraging human corrections, LLMs may further improve factual consistency and evidence-based reasoning. Furthermore, employing strategies such as human preference alignment and curriculum learning can help LLMs learn from their mistakes and progressively improve their performance in generating in-depth responses.

In the Supplementary Material, we show the specific prompt engineering templates (Supplementary Table A2), the reference question and answer pairs (Supplementary Table A3), and examples of AI-generated questions and answers (Supplementary Tables A4–A7). It can be noted that the AI-generated questions can closely emulate the text style of the reference questions to a significant extent. Although the reference answers are concise and short, it is likely a consequence of the LLMs' training on extensive dataset for general text generation tasks as well as medical question-answering, resulting in the generated answers containing substantial discussion and explanations of causes. Moreover, it can be observed that the LLMs, primarily trained on English corpora, occasionally produce answers in English (e.g., Supplementary Table A7). In addition, AI-generated questions that receive an overall low score may be attributed to the LLMs could omit several medical procedures or tests, thereby directly inquiring about advanced stages of the patient's treatment. This is a typical case of LLMs' hallucination issue. For instance, Hunyuan's question in the Supplementary Material Table A5 should not ask about “treatment” (the provided information does not substantiate a diagnosis for the patient, thus bypassing the diagnostic reasoning stage), but rather should focus on “what further tests should be conducted next”.

Our findings align with the broader literature on LLM-generated medical questions (14), which concludes that LLMs can produce valid exam items but require human oversight. Consistent with prior blinded comparison studies (16, 17), we found that LLM-generated questions approach human expert levels on surface-level quality metrics, while answer generation remains a greater challenge. Distinct from existing work that relies on textbook content and multiple-choice formats, our study extends this line of research to open-ended questions derived from real-world EHRs, reflecting the practical workflow of clinical exam development.

Limitations of this study: Generative models could produce contradictory information, as they are predominantly reliant on the extensive corpus and the probability distribution of the upcoming words during the autoregressive generation. Moreover, the prompt engineering method can stimulate LLMs' pre-trained knowledge within the medical domain and adapt text styles to resemble the reference, but it cannot guarantee the qualities once and for all. Consequently, the limited real-world reasoning abilities of LLMs can potentially result in false conclusions. Additionally, biases from references and prompting templates may yield discriminatory outputs, requiring human supervision. Efforts are needed to mitigate these biases through human supervision. Furthermore, LLM could inadvertently generate a response containing unique combination of symptoms, demographic, and treatment details, which can allow users to re-identify the patient. This issue could be alleviated by only providing the absolute minimum necessary data to LLMs required for the task and employing post-processing filters to detect high-risk combinations of information. Moreover, this study did not employ multimodal content and was conducted on database of Chinese elderly patients with chronic diseases, which might limit the generalizability of the model. Medical multimodal generative models is still in its early stages of development. The natural evolution of this work lies in harnessing multimodal data, combining EHRs with medical images, ECG waveforms, and clinical videos to generate comprehensive exam questions. Nevertheless, realizing this potential necessitates overcoming major challenges, such as developing sophisticated multimodal fusion techniques, creating large-scale annotated multimodal datasets, increasing the authenticity of AI-generated content evaluations, and addressing associated data privacy concerns.

Furthermore, Mistral 7B, a relatively small, English-predominant model, achieved the second-highest score in question generation. Mistral 7B demonstrated better instruction-following capability for the prompts, producing concise questions that closely matched the style of reference examples, which may have received higher ratings from evaluators. Furthermore, the evaluation focused on the overall quality metrics rather than in-depth reasoning depth, which may favor shorter, simpler outputs. This finding warrants further validation with larger sample sizes and more fine-grained clinical reasoning metrics in future studies.

This study primarily aimed to provide a practical benchmark of the current commercial LLM ecosystem for medical exam generation. It is important to emphasize that this study does not aim to conduct a rigorous performance comparison across different LLMs; our primary objective is to propose and explore a new direction for integrating LLMs into medical education. While the observed performance may be influenced by factors such as model scale and architecture, our focus remains on investigating the new paradigm rather than explaining or ranking inter-model differences. From an application-oriented perspective, this can represent the heterogeneous choices available to practitioners. Future work could involve controlled comparisons of LLMs with task-oriented pre-trained corpus, model scale and architecture. Additionally, the outputs were generated in a single run with each model's default temperature settings. The sampling stochasticity inherent to LLMs may introduce noise into individual item scores, and multiple generations per item could yield more stable performance estimates.

Furthermore, inter-rater reliability differs notably between the two evaluation tasks. Question generation yields higher ICC values, with overall average-rater ICC(2,3) reaching 0.810. This is expected as the task is anchored to source EHR content, with relatively objective criteria for factual accuracy and information completeness, facilitating consensus among evaluators. In contrast, open-ended answer generation shows substantially lower agreement [overall ICC(2,3) = 0.328]. For geriatric comorbidity cases, no single definitive gold standard exists for clinical reasoning; evaluators may hold varying thresholds for evidence adequacy, clinical priority, and expected level of detail.

The evaluation of medical exams was contingent upon humans. Due to the resource-intensive and time-consuming nature of having medical experts write comprehensive reviews, a scoring system was adopted. Besides, since this study employed competitive LLMs, we did not employ additional AI models as automatic evaluators. Furthermore, automated evaluation methods at the level of tokens, words, or phrases are dependent on the reference, and the cost associated with eliciting open-ended references from medical experts is prohibitively high. These factors highlight the constraints of evaluation phase, and also points to a potential avenue for the subsequent research.

Generating medical questions and answers with LLMs from EHRs shows significant potential for enhancing AI medical education. However, this approach necessitates careful ethical and privacy considerations. A primary risk involves the potential for models to memorize and inadvertently reveal sensitive patient information. To mitigate this, strict data de-identification protocols are essential. Furthermore, compliance with regulatory standards and ongoing oversight are crucial to ensure this technology is developed and applied responsibly, safeguarding patient privacy while harnessing its educational benefits.

## 5 Conclusion

This study extends prior research on LLM-generated medical assessment content to open-ended, EHR-based question and answer generation. Concretely, this study provides valuable insights into the application of LLMs for the generation of medical exam. LLMs showed performance approaching that of human experts on some evaluation aspects. Further, although LLMs showed promise in generating questions, there remains substantial scope for advancement in the quality of the answers. By harnessing the feedback of medical experts using few-shot prompt engineering, we hope to provide insights for refining LLMs in future work, thereby contributing to the ongoing exploration of how these models may assist in the medical education domain.

## Data Availability

The data that support the findings of this study are available on reasonable request from the corresponding author. Access to the dataset requires a formal application to the corresponding author and the CECMed data management committee, including a detailed research proposal. The dataset provided is de-identified and exclusively for non-commercial academic research. Commercial use, redistribution, resale, or public sharing of the data to unauthorized third parties is prohibited. All users must comply with the Declaration of Helsinki, medical ethics guidelines, and relevant national regulations on human genetic resources. Re-identification of study participants is strictly forbidden. The code used for this study is available at https://github.com/zhuyunqi96/medEduLLM.
