# Supplementary material for: The potential of LLMs in generating questions and answers with EHRs
Source: Front Digit Health. 2026 Jul 15;8:1799629. doi: 10.3389/fdgth.2026.1799629 (PMC13417121; doi:10.3389/fdgth.2026.1799629)
Supplement: Supplementary file 1 [file Datasheet1.pdf]

Appendix A

Table A1: Likert 5-point scale and evaluation metrics

1	2	3	4	5
Very poor	Poor	Adequate	Good	Excellent
Question evaluation				
**Coherence**: Does the question read fluently and is it logically structured ?				
**Sufficiency of Key Information**: Does the question contain the necessary context from the EHR to be answerable ?				
**Information Correctness**: Does the question accurately reflect the facts of the EHR case ?				
**Professionalism**: Is the terminology accurate and the style appropriate for a medical question ?				
Answer evaluation				
**Coherence**: Is the answer well-organized and easy to follow ?				
**Factual Consistency**: Is the medical information in the answer factually correct according to established knowledge ?				
**Evidence of Statement**: Does the answer provide reasoning or reference to evidence?				
**Professionalism**: Is the terminology accurate and the style appropriate for a medical answer ?				

Fig. A1: Statistics of patient records in the CECMed dataset.

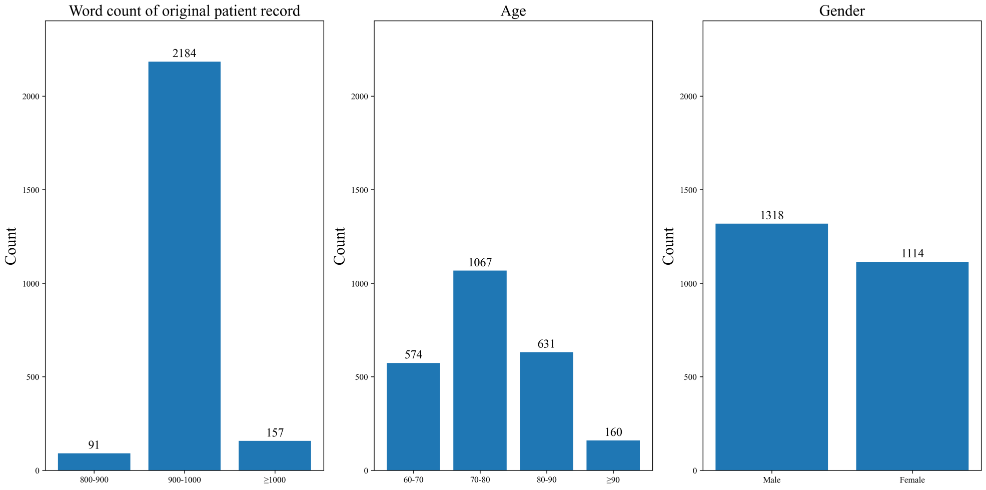

Table A2: Prompt Templates

Type	Prompt	Original Prompt (Chinese)
Question Generation	(System) You are an experienced clinical medical expert.<br>(User) Task: Refer to the following report sample and question sample to write a question for the patient's report.<br>Report Sample: #report-sample,<br>Question Sample: #question-sample,<br>Patient Report: #report-target,<br>Output Format:<br>"Question": ""	(System) 你是一个资深临床医学专家。<br>(User) 任务：参考以下报告样例与问题样例，为患者报告写一个提问。<br>报告样例：#report-sample<br>问题样例：#question-sample<br>患者报告：#report-target<br>输出格式："提问"：""
Answer Generation	(System) You are an experienced clinical medical expert.<br>(User) Task: Refer to the following question sample and answer sample to write an answer for the medical question.<br>Question Sample: #question-sample,<br>Answer Sample: #answer-sample,<br>Medical question: #question-target<br>Output Format:<br>"Answer": ""	(System) 你是一个资深临床医学专家。<br>(User) 任务：参考以下问题样例与答案样例，为医学问题写一个答案。<br>问题样例：#question-sample<br>答案样例：#answer-sample<br>医学问题：#question-target<br>输出格式："答案"：""
Answer revision	(System) You are an experienced clinical medical expert. Refer to the following question sample, answer sample and answer review.<br>Question Sample: #question-sample$_1$,<br>Answer Sample: #answer-sample$_1$,<br>Answer Review: #review-sample$_1$.<br>...<br>Question Sample: #question-sample$_n$[1],<br>Answer Sample: #answer-sample$_n$,<br>Answer Review: #review-sample$_n$.<br>(User) Task: Review the following medical question and answer to determine whether the answer needs to be corrected. If "yes", provide the corrected answer. If "no,"leave the answer blank.<br>Medical question: #question-target,<br>Medical answer: #answer-target,<br>Output Format:<br>"Whether it needs to be corrected": ""<br>"Answer": ""	(System) 你是一个资深临床医学专家。参考以下问题样例、答案样例和答案评价。<br>问题样例：#question-sample$_1$,<br>答案样例：#answer-sample$_1$,<br>答案评价：#review-sample$_1$.<br>...<br>问题样例：#question-sample$_n$[1],<br>答案样例：#answer-sample$_n$,<br>答案评价：#review-sample$_n$.<br>(User) 任务：检查以下医学问题和答案，判断是否需要纠正答案。如果"是"，请给出修正的答案，如果"否"，答案留空。<br>医学问题：#question-target,<br>医学答案：#answer-target,<br>输出格式：<br>"是否需要被纠正"：""<br>"Answer"：""

[1] Since a limited number of medical expert reviews (n=20) were collected for the corresponding questions and answers, concretely, we provided all n samples as the contextual background knowledge to the "system".

Table A3: Examples of reference questions and answers. Original Chinese texts are highlighted in red.

**Question**<br>A 70-year-old male with a history of hypertension, coronary heart disease, and reflux esophagitis presents with chest tightness and chest pain. The myocardial enzyme profile indicates a creatine kinase level of 291U/L and a troponin T level of 0.87ng/ml. What possible diagnoses should be considered? 一名 70 岁男性，既往有高血压、冠心病、反流性食管炎病史，此次出现胸闷、胸痛，心肌酶谱显示肌酸激酶为 291U/L，肌钙蛋白 T 为 0.87ng/ml，应考虑可能诊断为?<br><br>**Answer**<br>Acute Myocardial Infarction 急性心梗
**Question**<br>An 80-year-old male patient has been admitted due to chest pain, chest tightness, and shortness of breath after exertion. His blood pressure is 106/57mmHg, heart rate is 67 beats per minute, respiratory rate is 18 breaths per minute, and peripheral oxygen saturation reading is 97%. What checks should be conducted first? 80 岁男性，因胸痛、胸闷，活动后气短入院，血压 106/57mmHg，查体心率 67 次/分，呼吸 18 次/分，脉氧 97%。应首先完善什么检查。<br><br>**Answer**<br>Electrocardiogram, Myocardial Enzyme Profile 心电图，心肌酶谱
**Question**<br>A 67-year-old male patient with a history of diabetes mellitus and coronary heart disease has been admitted due to nausea and vomiting. Physical examination indicates an enlarged cardiac silhouette and lower limb edema. After admission, the creatine kinase level is found to be 200 U/L, and the troponin T level is 0.66 ng/ml. What additional diagnostic tests should be performed? 一名 67 岁男性，既往有糖尿病、冠心病病史，此次因恶心、呕吐入院，查体心界扩大，下肢有水肿，入院后查肌酸激酶为 200U/L，肌钙蛋白 T 为 0.66ng/ml，还应完善什么检查?<br><br>**Answer**<br>Urinalysis, NT-proBNP 尿常规，NT-proBNP
**Question**<br>A 71-year-old male patient presents with the main clinical symptoms of cough, expectoration, dyspnea, and fever. Phsical examination reveals there are moist rales audible in the lungs, edema in the lower limbs, cyanosis of the lips with a few wheezing sounds sounds. What additional additional tests should be performed? 一名 71 岁的男性，主要临床症状为咳嗽、咳痰、呼吸困难、发热，显示肺部可闻及湿罗音，下肢水肿，口唇紫绀，可闻及少量哮鸣音。应考虑完善哪些检查<br><br>**Answer**<br>Blood routine examination, Chest radiograph, BNP (Brain natriuretic peptide) 血常规，胸片，BNP

Table A4: Examples of AI-generated questions. Original Chinese texts are highlighted in red.

Report

1. Baseline Information: The patient is an 85-year-old female who has never smoked or consumed alcohol, with no history of insomnia or use of sleep aids, and no history of emergency visits or surgeries. Her height is 150 cm, weight is 55 kg, and BMI is 24.44. She has a medical history that includes hypertension, coronary atherosclerotic heart disease, hyperlipidemia, moderate to severe chronic kidney disease, and peptic ulcer disease. 1. 基线资料：患者为一名 85 岁的女性，从未抽烟和饮酒，无失眠和使用助眠药物史，无急诊入院和手术史。她的身高为 150 厘米，体重为 55 千克，BMI 为 24.44，有高血压、冠状动脉粥样硬化性心脏病、高脂血症、中重度慢性肾病和消化性溃疡等病史。

2. Vital Signs: The patient's body temperature is 36.9 degrees Celsius, blood pressure is 140 mmHg systolic and 66 mmHg diastolic, heart rate is 61 beats per minute, respiratory rate is 19 breaths per minute, and pulse oximetry is 96%. 2. 生命体征：患者的体温为 36.9 摄氏度，血压收缩压为 140 毫米汞柱，舒张压为 66 毫米汞柱，心率为 61 次/分钟，呼吸为 19 次/分钟，脉氧为 96%。

3. Current Symptoms and Signs: The patient's main manifestations include chest pain, palpitations, shortness of breath upon exertion, dizziness, and headaches. Clinically, there are no significant abnormalities in physical signs. 3. 目前症状和体征：患者主要表现为胸痛、心悸、活动后气短、头晕、头痛等症状。临床体征方面，无明显异常。

4. Comprehensive Geriatric Assessment: According to the Barthel score, the patient scores 90 points, indicating good self-care ability. The fall risk score is 60 points, suggesting the patient has a certain degree of balance impairment. 4. 老年综合评估：根据 Barthel 得分，患者评分为 90 分，表示生活自理能力良好。跌倒风险得分为 60 分，表明患者有一定的平衡障碍。

5. 5. Laboratory Examination: The complete blood count shows a neutrophil percentage of 57.9%, lymphocyte percentage of 28.8%, hemoglobin level of 127 g/L, and hematocrit of 38.4%. In the biochemical indicators, alanine aminotransferase is 7 mmol/L, aspartate aminotransferase is 16.4 mmol/L, total protein is 73.7 g/L, albumin is 41.1 g/L, total bilirubin is 6.89 mol/L, direct bilirubin is 3.75 mol/L, calcium ion concentration is 2.27 mg/L, potassium ion concentration is 4.31 mg/L, sodium ion concentration is 138.4 mg/L, chloride ion concentration is 109 mg/L, lactic acid is 1.78 mg/L, globulin is 32.6 g/L, and indirect bilirubin is 3.14 mol/L. In the DIC indicators, the international normalized ratio is within the normal range, fibrinogen is 3.57 g/L, and antithrombin III is 87.9%. In the myocardial enzyme profile, creatine kinase is 54 U/L, and troponin T is 0.01 ng/ml. In the urinalysis, ketone bodies, occult blood, leukocytes, and red blood cells are all normal, glucose is 5.58 mg/dL, and the pH is 6. 5. 实验室检查：血常规显示中性粒细胞百分比为 57.9%，淋巴细胞百分比为 28.8%，血红蛋白为 127 克/升，红细胞压积为 38.4%。生化指标中，谷丙转氨酶为 7 毫摩尔/升，谷草转氨酶为 16.4 毫摩尔/升，总蛋白为 73.7 克/升，白蛋白为 41.1 克/升，总胆红素为 6.89 微摩尔/升，直接胆红素为 3.75 微摩尔/升，钙离子浓度为 2.27 毫克/升，钾离子浓度为 4.31 毫克/升，钠离子浓度为 138.4 毫克/升，氯离子浓度为 109 毫克/升，乳酸为 1.78 毫克/升，球蛋白为 32.6 克/升，间接胆红素为 3.14 微摩尔/升。DIC 指标中，国际标准化比值处于正常范围，纤维蛋白原为 3.57 克/升，抗凝血酶 III 为 87.9%。心肌酶谱中，肌酸激酶为 54U/L，肌钙蛋白 T 为 0.01ng/ml。尿常规中，酮体、潜血、白细胞和红细胞均正常，葡萄糖为 5.58mg/dL，酸碱度为 6。

Questions

An 85-year-old female patient with a history of hypertension, coronary heart disease, hyperlipidemia, and moderate to severe chronic kidney disease presents with symptoms of chest pain, palpitations, and shortness of breath upon exertion. Laboratory examination reveals a troponin T level of 0.01 ng/ml. How should further diagnosis and treatment be approached? 85 岁女性患者，有高血压、冠心病、高脂血症和中重度慢性肾病等病史，目前出现胸痛、心悸、活动后气短等症状，实验室检查发现肌钙蛋白 T 为 0.01ng/ml，应如何进一步诊断和治疗?
Coherence: 5, Sufficiency of key information: 3, Information correctness: 5, Professionalism: 5 (ERNIE 4)

An 85-year-old female patient with a medical history of hypertension, coronary heart disease, hyperlipidemia, chronic kidney disease, and peptic ulcer disease is currently experiencing chest pain, palpitations, shortness of breath after exertion, dizziness, and headaches. Her creatine kinase level is 54 U/L, and troponin T is 0.01 ng/ml. To further clarify the diagnosis, what additional examinations should be conducted? 一名 85 岁女性患者，有高血压、冠心病、高脂血症、慢性肾病和消化性溃疡病史，目前出现胸痛、心悸、活动后气短、头晕、头痛等症状，肌酸激酶为 54U/L，肌钙蛋白 T 为 0.01ng/ml。为进一步明确诊断，还应进行哪些检查?
Coherence: 2, Sufficiency of key information: 3, Information correctness: 5, Professionalism: 5 (ChatGLM 4)

An 85-year-old female with a history of hypertension, coronary heart disease, hyperlipidemia, moderate to severe chronic kidney disease, and peptic ulcer disease is presenting with symptoms of chest pain, palpitations, shortness of breath after exertion, dizziness, and headaches. Having completed the aforementioned examinations, what special investigations are needed to identify the etiology? 一名 85 岁女性，有高血压、冠心病、高脂血症、中重度慢性肾病和消化性溃疡病史，目前有胸痛、心悸、活动后气短、头晕、头痛等症状，已完善上述检查，还需要进行哪些特殊检查来明确病因?
Coherence: 2, Sufficiency of key information: 2, Information correctness: 2, Professionalism: 2 (Doubao)

An 85-year-old female with a history of hypertension, coronary heart disease, hyperlipidemia, moderate to severe chronic kidney disease, and peptic ulcer disease reports chest pain, palpitations, shortness of breath after exertion, dizziness, and headaches. Her myocardial enzyme profile shows creatine kinase at 54 U/L and troponin T at 0.01 ng/ml. What further examinations should be conducted to assess her condition? 一名 85 岁女性，有高血压、冠心病、高脂血症、中重度慢性肾病和消化性溃疡病史，主诉胸痛、心悸、活动后气短、头晕、头痛，心肌酶谱中肌酸激酶为 54U/L，肌钙蛋白 T 为 0.01ng/ml，还应进行哪些检查以进一步评估其病情?
Coherence: 4, Sufficiency of key information: 2, Information correctness: 5, Professionalism: 4 (Hunyuan)

An 85-year-old female with a past medical history of hypertension, coronary heart disease, hyperlipidemia, moderate to severe chronic kidney disease, and peptic ulcer disease has been admitted due to chest pain, palpitations, shortness of breath after exertion, dizziness, and headaches. Physical examination reveals no significant abnormalities, and her myocardial enzyme profile shows creatine kinase at 54 U/L and troponin T at 0.01 ng/ml. What additional examinations should be completed? 一名 85 岁女性，既往有高血压、冠心病、高脂血症、中重度慢性肾病和消化性溃疡等病史，此次因胸痛、心悸、活动后气短、头晕、头痛入院，查体无明显异常，心肌酶谱中肌酸激酶为 54U/L，肌钙蛋白 T 为 0.01ng/ml，还应完善什么检查?
Coherence: 5, Sufficiency of key information: 4, Information correctness: 5, Professionalism: 5 (Llama 3 70B)

Based on the patient's symptoms and laboratory examination results, what further tests should be conducted to determine potential visceral injury or other abnormalities? 根据患者的症状和实验室检查结果，应进一步检查什么项目来确定患者可能存在的内脏损伤或其他异常?
Coherence: 4, Sufficiency of key information: 3, Information correctness: 3, Professionalism: 4 (Mistral 7B)

An 85-year-old female with a history of hypertension, coronary heart disease, and chronic kidney disease presents with chest pain, palpitations associated with shortness of breath after exertion. Laboratory tests indicate a creatine kinase level of 54 U/L and troponin T of 0.01 ng/ml. To further diagnose and assess the condition, what tests should be conducted next for diagnosis? 一名 85 岁女性，有高血压、冠心病、慢性肾病等病史，主诉胸痛、心悸伴活动后气短，实验室检查肌酸激酶为 54U/L，肌钙蛋白 T 为 0.01ng/ml，为进一步诊断和评估病情，应考虑进行哪些辅助检查?
Coherence: 5, Sufficiency of key information: 4, Information correctness: 5, Professionalism: 5 (Qwen)

An 85-year-old female with a medical history of hypertension, coronary atherosclerotic heart disease, hyperlipidemia, moderate to severe chronic kidney disease, and peptic ulcer disease is now exhibiting symptoms of chest pain, palpitations, shortness of breath after exertion, dizziness, and headaches. Her myocardial enzyme profile shows creatine kinase at 54 U/L and troponin T at 0.01 ng/ml. What diseases should be considered based on the above findings? 85 岁女性，有高血压、冠状动脉粥样硬化性心脏病、高脂血症、中重度慢性肾病和消化性溃疡等病史，现表现为胸痛、心悸、活动后气短、头晕、头痛，心肌酶谱中肌酸激酶为 54U/L，肌钙蛋白 T 为 0.01ng/ml。请问这些症状和检查结果可能提示哪些疾病?
Coherence: 4, Sufficiency of key information: 3, Information correctness: 5, Professionalism: 5 (Spark 4)

Table A5: Examples of AI-generated questions. Original Chinese texts are highlighted in red.

Report

1. Baseline Information: The patient is a 67-year-old female who has No history of smoking or alcohol consumption, denies insomnia and the use of sleep aids, has no history of emergency admissions or surgeries, stands at a height of 167 cm, weighs 68 kg, with a BMI of 24.38, a waist circumference of 101 cm. She has been enrolled with five conditions, primarily due to chest pain. Diagnoses include coronary atherosclerotic heart disease, hypertension stage 3 (very high risk), hyperlipidemia, and type 2 diabetes mellitus. 1. 基线资料：患者为女性，67 岁，已戒 1 年以上从未抽烟，无吸烟数量和年限，已戒 1 年以上从未饮酒，无吸烟饮酒史，无失眠和使用助眠药物，无急诊入院和手术史，167cm 身高，68kg 体重，24.38 BMI，101cm 腰围，入组五种疾病，主要原因为胸痛，诊断包括冠状动脉粥样硬化性心脏病、高血压病 3 级（很高危）、高脂血症和 2 型糖尿病。

2. Vital Signs: The patient's body temperature is 36.3°C, blood pressure is 118/66 mmHg, and heart rate is 77 beats per minute. 2. 生命体征：患者的体温为 36.3℃，血压为 118/66mmHg，心率为 77 次/分。

3. Current Symptoms and Signs: The patient's main clinical symptom is chest pain, with no other obvious symptoms or signs. 3. 目前症状和体征：患者主要临床症状为胸痛，无其他明显症状和体征。

4. Comprehensive Geriatric Assessment: The Barthel score is 100 points, present with chronic pain, and the fall risk score is 45.. 4. 老年综合评估：Barthel 得分为 100 分，有慢性疼痛，跌倒风险得分为 45 分。

5. Laboratory Examination: Blood routine shows a neutrophil percentage of 55%, lymphocyte percentage of 37.3%, hemoglobin at 127 g/L, hematocrit at 38.9%, mean corpuscular hemoglobin content at 28.9 pg, platelets at 203×10^9/L, mean platelet volume at 9.4 fl. Biochemical indicators include alanine aminotransferase at 16 U/L, aspartate aminotransferase at 15.1 U/L, total protein at 75.3 g/L, albumin at 45 g/L, total bilirubin at 14.99 umol/L, direct bilirubin at 6.26 umol/L, calcium ion at 2.34 mmol/L, potassium ion at 4.07 mmol/L, sodium ion at 139.2 mmol/L, chloride ion at 105 mmol/L, lactate at 3.01 mmol/L, globulin at 30.3 g/L, indirect bilirubin at 8.73 umol/L. DIC-related indicators are normal. In the myocardial enzyme profile, creatine kinase is 78 U/L, and troponin T is 0.01 ng/ml. Urinalysis shows normal ketone bodies, occult blood, leukocytes, and red blood cells, with glucose at 11.4 mg/dL. 5. 实验室检查：血常规显示中性粒细胞百分比为 55%，淋巴细胞百分比为 37.3%，血红蛋白为 127g/L，红细胞压积为 38.9%，平均红细胞血红蛋白含量为 28.9pg，血小板为 203×10^9/L，平均血小板体积为 9.4fl，生化指标中谷丙转氨酶为 16U/L，谷草转氨酶为 15.1U/L，总蛋白为 75.3g/L，白蛋白为 45g/L，总胆红素为 14.99umol/L，直接胆红素为 6.26umol/L，钙离子为 2.34mmol/L，钾离子为 4.07mmol/L，钠离子为 139.2mmol/L，氯离子为 105mmol/L，乳酸为 3.01mmol/L，球蛋白为 30.3g/L，间接胆红素为 8.73umol/L，DIC 相关指标正常，心肌酶谱中肌酸激酶为 78U/L，肌钙蛋白 T 为 0.01ng/ml。尿常规示酮体、潜血、白细胞和红细胞均正常，葡萄糖为 11.4mg/dL。

Questions

A 67-year-old female patient was admitted to the hospital due to chest pain and has been diagnosed with coronary atherosclerotic heart disease, hypertension stage 3 (very high risk), hyperlipidemia, and type 2 diabetes mellitus. Currently, her vital signs are stable. Which examinations should be prioritized for completion? 67 岁女性患者，因胸痛入院，诊断为冠状动脉粥样硬化性心脏病、高血压病 3 级（很高危）、高脂血症和 2 型糖尿病，目前生命体征平稳，应首先完善哪些检查?
Coherence: 5, Sufficiency of key information: 5, Information correctness: 5, Professionalism: 5 (ERNIE 4)

A 67-year-old female patient was admitted to the hospital with chest pain, presenting with a blood pressure of 118/66 mmHg and a heart rate of 77 beats per minute. Based on the patient's condition, which examination should be prioritized to assist in the diagnosis? 67 岁女性患者，因胸痛入院，血压 118/66mmHg，心率为 77 次/分。根据患者情况，应首先完善哪项检查以协助诊断?
Coherence: 5, Sufficiency of key information: 2, Information correctness: 5, Professionalism: 4 (ChatGLM 4)

A 67-year-old female was admitted to the hospital due to chest pain and has been diagnosed with multiple conditions. Her blood pressure is 118/66 mmHg, heart rate is 77 beats per minute, and laboratory examination reveals a lactate level of 3.01 mmol/L, which is elevated. What could be the possible causes? 67 岁女性，因胸痛入院，诊断有多种疾病，血压 118/66mmHg，心率 77 次/分，实验室检查乳酸为 3.01mmol/L 偏高，可能的原因是什么?
Coherence: 5, Sufficiency of key information: 5, Information correctness: 5, Professionalism: 5 (Doubao)

A 67-year-old female patient was admitted to the hospital with chest pain and has been diagnosed with coronary atherosclerotic heart disease, hypertension stage 3 (very high risk), hyperlipidemia, and type 2 diabetes mellitus. Her blood pressure is 118/66 mmHg, and her heart rate is 77 beats per minute. In the current clinical scenario, which treatment measures should be prioritized? 67 岁女性患者，因胸痛入院，已诊断为冠状动脉粥样硬化性心脏病、高血压病 3 级（很高危）、高脂血症和 2 型糖尿病。她的血压为 118/66mmHg，心率为 77 次/分。在目前的临床情况下，应优先考虑哪些治疗措施?
Coherence: 1, Sufficiency of key information: 1, Information correctness: 5, Professionalism: 1 (Hunyuan)

A 67-year-old female was admitted to the hospital with chest pain, and her physical examination reveals a blood pressure of 118/66 mmHg and a heart rate of 77 beats per minute. What examination should be prioritized for completion? 67 岁女性，因胸痛入院，血压 118/66mmHg，查体心率 77 次/分。应首先完善什么检查。
Coherence: 4, Sufficiency of key information: 4, Information correctness: 5, Professionalism: 3 (Llama 3 70B)

What tests should be performed to assess the patient's thrombotic risk? Which examination should be completed first? 应完善何种检查评估患者血栓风险，应先完善什么检查?
Coherence: 3, Sufficiency of key information: 3, Information correctness: 5, Professionalism: 5 (Mistral 7B)

For a 67-year-old female patient with a chief complaint of chest pain and a diagnosis that includes coronary atherosclerotic heart disease, currently presenting with a blood pressure of 118/66 mmHg and a heart rate of 77 beats per minute, what further examination should be considered first to evaluate the cause of chest pain? 针对 67 岁女性患者，主诉胸痛，且诊断包含冠状动脉粥样硬化性心脏病等，目前血压 118/66mmHg，心率 77 次/分，首先应考虑进行什么进一步的检查来评估胸痛原因?
Coherence: 3, Sufficiency of key information: 3, Information correctness: 4, Professionalism: 4 (Qwen)

A 67-year-old female was admitted to the hospital with chest pain and has a history of diagnoses including coronary atherosclerotic heart disease, hypertension stage 3 (very high risk), hyperlipidemia, and type 2 diabetes mellitus. Current blood routine and biochemical indicators show a neutrophil percentage of 55%, hemoglobin at 127 g/L, alanine aminotransferase at 16 U/L, aspartate aminotransferase at 15.1 U/L, total protein at 75.3 g/L, and albumin at 45 g/L. Are these indicators within the normal range? 67 岁女性，因胸痛入院，既往诊断为冠状动脉粥样硬化性心脏病、高血压病 3 级（很高危）、高脂血症和 2 型糖尿病。目前血常规和生化指标显示中性粒细胞百分比为 55%，血红蛋白为 127g/L，谷丙转氨酶为 16U/L，谷草转氨酶为 15.1U/L，总蛋白为 75.3g/L，白蛋白为 45g/L。请问这些指标是否在正常范围内?
Coherence: 5, Sufficiency of key information: 4, Information correctness: 5, Professionalism: 5 (Spark 4)

Table A6: Examples of AI-generated answers. Original Chinese texts are highlighted in red.

Question

A 78-year-old female with a medical history significant for hypertension, coronary heart disease, diabetes mellitus, hyperlipidemia, and glaucoma presented with symptoms of shortness of breath and fatigue. Laboratory findings revealed a hemoglobin level of 107g/L, hematocrit at 33.1%, urine glucose of 12.35mg/dL, lactic acid at 1.82mmol/L, creatine kinase of 102U/L, and troponin T at 0.23ng/ml. Please consider the possible diagnoses and provide an explanation for the findings. 一名 78 岁女性，既往有高血压、冠心病、糖尿病、高脂血症和青光眼病史，此次出现憋气和乏力，血常规显示血红蛋白 107g/L，红细胞压积 33.1%，尿常规中葡萄糖 12.35mg/dL，乳酸 1.82mmol/L，肌酸激酶 102U/L，肌钙蛋白 T0.23ng/ml。请考虑可能诊断为何，并解释原因？

Answers

The possible diagnoses are diabetic cardiomyopathy and chronic cardiac insufficiency. Diabetic Cardiomyopathy: The patient has a long-standing history of diabetes mellitus, which can lead to alterations in the microstructure and function of the myocardium, thereby affecting cardiac systolic and diastolic function. Diabetic cardiomyopathy is commonly characterized by cardiac hypertrophy, heart failure, and arrhythmias. The patient's symptoms of fatigue and shortness of breath, along with a mild elevation of troponin T, suggest the possibility of myocardial injury. Chronic Cardiac Insufficiency: The patient's history of hypertension and coronary heart disease is significant, as these are major precipitating factors for cardiac insufficiency. Chronic hypertension can result in increased afterload, leading to myocardial hypertrophy and cardiac dilation. Coronary heart disease can cause myocardial ischemia, impacting cardiac contractility. Additionally, the patient's blood tests show slightly low hemoglobin and hematocrit levels, which may reflect hemodilution or mild anemia, and anemia can exacerbate the symptoms of cardiac insufficiency. The patient's symptoms of fatigue and shortness of breath are likely due to cardiac insufficiency. In summary, considering the patient's medical history, symptoms, and laboratory findings, the diagnoses of diabetic cardiomyopathy and chronic cardiac insufficiency are proposed. It is important to note that this is a preliminary assessment based on the current information, and a definitive diagnosis requires further clinical data and comprehensive evaluation by a healthcare professional. 可能诊断为糖尿病性心肌病和慢性心功能不全。1. ** 糖尿病性心肌病 **：患者长期患有糖尿病，这可能导致心肌的微观结构和功能改变，从而影响心脏的收缩和舒张功能。糖尿病性心肌病常表现为心脏肥大、心力衰竭和心律失常。患者的乏力、憋气症状，以及肌钙蛋白 T 的轻度升高，均提示可能存在心肌损伤。2. ** 慢性心功能不全 **：患者既往有高血压、冠心病病史，这些都是心功能不全的重要诱因。长期的高血压可导致心脏后负荷增加，引起心肌肥厚和心脏扩大；而冠心病可导致心肌缺血，影响心脏的收缩功能。此外，患者的血常规显示血红蛋白和红细胞压积略低，这可能反映了血液稀释或轻度贫血，而贫血也可能加重心功能不全的症状。患者的乏力和憋气症状，很可能是心功能不全导致的。综上所述，结合患者的病史、症状和实验室检查，考虑诊断为糖尿病性心肌病和慢性心功能不全。需要注意的是，这只是基于当前信息的初步判断，具体的诊断还需要结合更多的临床资料和医生的综合评估。

Coherence: 3, Factual consistency: 3, Evidence of statement: 3, Professionalism: 2 (ERNIE 4)

The clinical presentation and examination findings of this patient may suggest several diagnostic possibilities: Heart Failure: Given the patient's history of hypertension and coronary heart disease, symptoms of shortness of breath and fatigue may be indicative of cardiac insufficiency. The mild elevation of troponin T may suggest myocardial injury. Anemia: The below-normal levels of hemoglobin and hematocrit may indicate the presence of anemia, which could lead to fatigue and other systemic symptoms. Complications of Diabetes: The significantly elevated glucose in the urine suggests poor glycemic control, which may be due to worsening diabetes or complications such as diabetic nephropathy. Metabolic Acidosis: The elevated lactate level may indicate the presence of metabolic acidosis, which could be due to diabetic ketoacidosis or lactic acidosis. Explanation of Causes: Heart Failure: Hypertension and coronary heart disease can lead to alterations in cardiac structure and function, resulting in heart failure. Anemia: This may be due to anemia of chronic disease associated with a chronic illness, or other causes such as malnutrition or bleeding. Complications of Diabetes: Prolonged hyperglycemia can result in kidney damage, leading to elevated glucose in the urine. Metabolic Acidosis: The increased lactate may be due to tissue hypoxia or diabetic ketoacidosis, which may be related to poor diabetes control. Considering the patient's medical history and examination results, it is recommended to conduct further investigations and assessments, including echocardiography, renal function tests, monitoring of blood glucose and glycated hemoglobin, as well as related examinations for metabolic acidosis, to establish a definitive diagnosis and develop an appropriate treatment plan. Please note that actual diagnosis and treatment should be performed by healthcare professionals after a detailed evaluation of the patient's condition. 该患者的临床表现和检查结果可能提示以下几种诊断：1. 心力衰竭：患者有高血压和冠心病病史，出现憋气和乏力可能是心功能不全的表现。肌钙蛋白 T 的轻度升高可能提示心肌损伤。2. 贫血：血红蛋白和红细胞压积低于正常值，可能表明患者存在贫血，这可能导致乏力和其他全身症状。3. 糖尿病并发症：尿常规中葡萄糖显著升高，提示血糖控制不佳，可能是糖尿病的加重或并发症，如糖尿病肾病。4. 代谢性酸中毒：乳酸水平升高可能表明存在代谢性酸中毒，这可能是由于糖尿病酮症酸中毒或乳酸酸中毒。原因解释：- 心力衰竭：高血压和冠心病可能导致心脏结构和功能的改变，从而引起心力衰竭。- 贫血：可能是由于慢性疾病引起的慢性病贫血，或是营养不良、出血等其他原因。- 糖尿病并发症：长期高血糖可能导致肾脏损伤，进而引起尿中葡萄糖升高。- 代谢性酸中毒：乳酸升高可能是由于组织缺氧或糖尿病酮症酸中毒，这可能与糖尿病控制不佳有关。综合考虑患者的病史和检查结果，建议进行进一步的检查和评估，包括心脏超声、肾功能检查、血糖和糖化血红蛋白监测，以及可能的代谢性酸中毒的相关检查，以确定最终诊断并制定相应的治疗方案。请注意，实际诊断和治疗应由专业医疗人员在详细评估患者状况后进行。

Coherence: 3, Factual consistency: 3, Evidence of statement: 3, Professionalism: 3 (ChatGLM 4)

The possible diagnosis is myocardial infarction, with concurrent diabetes-related metabolic disorders. The reasons are as follows: The patient has a history of coronary heart disease and presents with symptoms of shortness of breath and fatigue. The elevated troponin T level (0.23ng/ml, normally less than 0.1ng/ml) is a specific marker for the diagnosis of myocardial infarction. An increased troponin T level strongly suggests myocardial cell damage, making myocardial infarction a likely diagnosis. The patient has a history of diabetes mellitus, and the urine test showing glucose at 12.35mg/dL indicates poor glycemic control and the presence of diabetes-related metabolic abnormalities. The patient's hemoglobin level is slightly reduced at 107g/L (the normal range for adult females is generally 110 - 150g/L), and the hematocrit is slightly reduced at 33.1% (the normal range for females is approximately 37% - 48%), which may be associated with a chronic disease state but is not the primary focus of the current diagnosis. The slightly elevated lactate level at 1.82mmol/L may be related to inadequate tissue perfusion due to myocardial infarction. The creatine kinase level is within the normal range at 102U/L (the normal range for males is 50 - 310U/L, and for females, it is 40 - 200U/L). Although creatine kinase may increase during a myocardial infarction, troponin T is more specific and sensitive for the diagnosis of myocardial infarction. Additionally, the patient's history of glaucoma is unrelated to the current symptoms of shortness of breath and fatigue. 答案：可能的诊断为心肌梗死，同时存在糖尿病相关的代谢紊乱。原因如下：1. 患者既往有冠心病病史，此次出现憋气和乏力症状，肌钙蛋白 T 升高（0.23ng/ml，正常一般小于 0.1ng/ml），肌钙蛋白 T 是诊断心肌梗死的特异性指标，升高高度提示心肌细胞受损，很可能存在心肌梗死。2. 患者有糖尿病病史，尿常规中葡萄糖 12.35mg/dL 提示血糖控制不佳，存在糖尿病相关的代谢异常。3. 患者血红蛋白 107g/L（成年女性正常范围一般为 110 - 150g/L）轻度降低、红细胞压积 33.1%（正常女性约为 37% - 48%）轻度降低，可能与慢性疾病状态有关，但不是此次主要诊断方向。乳酸 1.82mmol/L 轻度升高可能与心肌梗死导致的组织灌注不足有关。肌酸激酶 102U/L（正常男性为 50 - 310U/L，女性为 40 - 200U/L）处于正常范围，虽然肌酸激酶在心肌梗死时可能升高，但肌钙蛋白 T 对心肌梗死诊断更特异且更敏感。此外，患者的青光眼病史与此次出现的憋气和乏力等症状无关。

Coherence: 4, Factual consistency: 4, Evidence of statement: 4, Professionalism: 4 (Doubao)

The patient's possible diagnosis is Acute Coronary Syndrome (ACS), for the following reasons: Characteristics of Medical History: Hypertension, coronary heart disease, diabetes mellitus, and hyperlipidemia are all risk factors for cardiovascular disease. Although glaucoma is not directly associated with cardiovascular disease, it suggests the presence of chronic diseases in other systems. Symptom Analysis: Shortness of breath and fatigue are common clinical manifestations of ACS, which may be due to decreased cardiac function resulting from myocardial ischemia. Laboratory Examination Results: Hemoglobin at 107g/L and hematocrit at 33.1% suggest mild anemia, which may be related to chronic disease but is not sufficient to explain the current symptoms. Glucose at 12.35mg/dL in the urine indicates the presence of diabetes mellitus and poor glycemic control. Lactate at 1.82mmol/L is slightly elevated, which may reflect tissue hypoxia or metabolic abnormality. Creatine kinase at 102U/L is slightly elevated, which may be associated with myocardial injury. Troponin T at 0.23ng/ml is significantly elevated, a specific marker for myocardial injury, strongly suggesting myocardial cell damage, commonly seen in ACS. In summary, considering the patient's medical history, symptoms, and laboratory examination results, the most likely diagnosis is Acute Coronary Syndrome. It is recommended to perform further examinations, such as an electrocardiogram and echocardiography, to confirm the diagnosis and to initiate appropriate treatment promptly. 该患者可能诊断为急性冠状动脉综合征（ACS），原因如下：1. ** 病史特点 **：- 高血压、冠心病、糖尿病和高脂血症均为心血管疾病的危险因素。- 青光眼虽与心血管疾病无直接关联，但提示患者可能存在其他系统的慢性疾病。2. ** 症状分析 **：- 憋气和乏力是 ACS 的常见临床表现，可能由于心肌缺血导致心功能下降。3. ** 实验室检查结果 **：- 血红蛋白 107g/L 和红细胞压积 33.1% 提示轻度贫血，可能与慢性疾病相关，但不足以解释当前症状。- 尿常规中葡萄糖 12.35mg/dL 表明患者存在糖尿病，且血糖控制不佳。- 乳酸 1.82mmol/L 略高于正常值，可能反映组织缺氧或代谢异常。- 肌酸激酶 102U/L 轻度升高，可能与心肌损伤有关。- 肌钙蛋白 T0.23ng/ml 明显升高，这是心肌损伤的特异性标志物，强烈提示心肌细胞受损，常见于 ACS。综上所述，结合患者的病史、症状和实验室检查结果，最可能的诊断为急性冠状动脉综合征。建议进一步行心电图、心脏超声等相关检查以明确诊断，并及时给予相应治疗。

Coherence: 4, Factual consistency: 3, Evidence of statement: 3, Professionalism: 3 (Hunyuan)

Based on the patient's medical history and laboratory findings, the possible diagnosis is Acute Heart Failure. Rationale: The patient's history of hypertension, coronary heart disease, and hyperlipidemia increases the risk for heart failure. The presence of symptoms such as shortness of breath and fatigue suggests the possibility of cardiac insufficiency. The blood test reveals hemoglobin at 107g/L and hematocrit at 33.1%, indicating no significant anemia, but heart failure cannot be ruled out. The urine test showing glucose at 12.35mg/dL suggests poor diabetes control, which may elevate the risk of heart failure. The slightly elevated levels of lactate at 1.82mmol/L and creatine kinase at 102U/L may indicate myocardial damage or heart failure. The mild elevation of troponin T to 0.23ng/ml also supports the potential diagnosis of myocardial damage or heart failure. In conclusion, considering the patient's medical history and laboratory results, acute heart failure is the most likely diagnosis; however, further examinations and assessments are required for confirmation. 基于该患者的医疗史和实验室结果，可能诊断为急性心力衰竭（Acute Heart Failure）。理由：1. 患者既往有高血压、冠心病和高脂血症的病史，增加了心力衰竭的风险。2. 患者出现憋气和乏力，提示可能存在心脏功能不全。3. 血常规显示血红蛋白 107g/L 和红细胞压积 33.1%，表明无明显贫血，但也不能排除心力衰竭的可能。4. 尿常规中葡萄糖 12.35mg/dL 表明患者的糖尿病控制不佳，可能增加了心力衰竭的风险。5. 乳酸 1.82mmol/L 和肌酸激酶 102U/L 的轻度升高可能提示心肌损害或心力衰竭。6. 肌钙蛋白 T0.23ng/ml 的轻度升高也支持心肌损害或心力衰竭的可能诊断。总之，考虑到患者的医疗史和实验室结果，急性心力衰竭是最可能的诊断，但需要进一步的检查和评估来确诊。

Coherence: 3, Factual consistency: 3, Evidence of statement: 3, Professionalism: 3 (Llama 3 70B)

In this scenario, the elevated hemoglobin level, high hematocrit, increased creatine kinase level, elevated creatine dehydration compound level, along with symptoms of shortness of breath and fatigue, may suggest a diagnosis of acidosis, potentially due to renal dysfunction leading to chronic kidney disease (CKD) or acute kidney injury (AKI). Additionally, hyperglycemia may contribute to the formation of pustular protein vesicles, increasing the hemoglobin level. The elevated creatine kinase level may be a result of muscle rupture or may be secondary to acidosis. A low troponin level might be indicative of renal dysfunction. 这种情况下，血红蛋白水平高、红细胞压积高、肌酸激酶水平高、肌酸脱水化合物水平高，以及出现憋气和乏力等症状，可能诊断为 $[UNK][UNK]$idosis（酸碱不平衡），可能是由于肾功能障碍导致的肾衰竭（Chronic Kidney Disease, CKD）或急性肾功能障碍（Acute Kidney Injury, AKI）。此外，高血糖水平可能会导致脓蛋白浆泡形成，增加血红蛋白水平。肌酸激酶水平高可能是肌肉破裂导致的，也可能是 $[UNK][UNK]$idosis 引起的。肌钙蛋白水平低可能是肾功能障碍导致的。

Coherence: 3, Factual consistency: 3, Evidence of statement: 3, Professionalism: 3 (Mistral 7B)

The 78-year-old female patient presents with symptoms of shortness of breath and fatigue. In consideration of her medical history (hypertension, coronary heart disease, diabetes mellitus, hyperlipidemia, and glaucoma) and laboratory examination results, several possible diagnoses should be considered: Acute Coronary Syndrome (such as unstable angina or myocardial infarction): Given the patient's history of coronary heart disease, shortness of breath and fatigue are typical symptoms of cardiac ischemia. Although the troponin T level is slightly elevated (0.23ng/ml), which may suggest minor myocardial damage, it is important to note that troponin elevation in elderly patients can be subtle. The creatine kinase level is not significantly elevated, but its assessment should be approached with caution in elderly patients due to atypical presentations. Anemia: Hemoglobin at 107g/L and hematocrit at 33.1% indicate mild anemia, which could be one of the causes of fatigue. In the elderly, anemia may be associated with chronic disease, result from malnutrition or chronic inflammation, and could also affect cardiac function, exacerbating symptoms of shortness of breath. Diabetic Complications: Glucose at 12.35mg/dL in the urine suggests poor glycemic control. Chronic hyperglycemia can lead to diabetic nephropathy, neuropathy, and other complications that may indirectly cause fatigue. Moreover, the hyperglycemic state itself can cause osmotic diuresis, leading to dehydration and electrolyte imbalance, which can impact cardiac function. Elevated Lactate Level: Lactate at 1.82mmol/L is slightly above the normal range (typically 0.5-1.6mmol/L), which may indicate tissue hypoperfusion or metabolic abnormality, conditions that can be seen in cardiovascular disease, severe anemia, or as a side effect of certain medications. In this patient's case, the elevated lactate level may be related to cardiac insufficiency and requires exclusion of other potential causes of metabolic acidosis. In summary, the patient's symptoms and laboratory findings point towards cardiovascular system issues, particularly acute coronary syndrome, compounded by the impact of mild anemia and diabetic complications. The elevated lactate level suggests a need for further evaluation of the metabolic state and tissue perfusion. Treatment should urgently address the cardiovascular condition, while also adjusting diabetes management, improving anemia, and identifying the exact cause of the elevated lactate levels. 该名 78 岁女性患者出现憋气和乏力的症状，结合其既往病史（高血压、冠心病、糖尿病、高脂血症和青光眼）及实验室检查结果，需要考虑以下几个可能的诊断：1. ** 急性冠脉综合征（如不稳定型心绞痛或心肌梗死）**：患者有冠心病史，憋气和乏力是心脏供血不足的典型症状。虽然肌钙蛋白 T 水平轻度升高（0.23ng/ml），这可能提示轻微的心肌损伤，但需注意的是，在老年患者中肌钙蛋白的升高可能较为微妙。肌酸激酶虽未显著升高，但考虑到非典型表现，在老年患者中应谨慎评估。2. ** 贫血 **：血红蛋白 107g/L 和红细胞压积 33.1% 表明存在轻度贫血，这可能是导致乏力的原因之一。在老年人中，贫血可能与慢性疾病相关，或是营养不良、慢性炎症等的结果，也可能影响心脏功能，加重憋气症状。3. ** 糖尿病并发症 **：尿常规中葡萄糖 12.35mg/dL 提示血糖控制不佳，长期高血糖可能导致糖尿病肾病、神经病变等并发症，这些都可能间接引起乏力。此外，高血糖状态本身也可引起渗透性利尿，导致脱水和电解质失衡，影响心脏功能。4. ** 乳酸水平升高 **：乳酸 1.82mmol/L 略高于正常范围（通常为 0.5-1.6mmol/L），可能提示组织灌注不足或代谢异常，这在心血管疾病、严重贫血或某些药物副作用中可见。在该患者情况下，乳酸升高可能与心脏功能不全有关，也需排除其他潜在的代谢性酸中毒原因。综上所述，该患者的症状和实验室检查结果指向了心血管系统的问题，特别是急性冠脉综合征，同时合并轻度贫血和糖尿病并发症的影响。乳酸水平升高提示需进一步评估代谢状态和组织灌注情况。治疗上应紧急处理心血管状况，同时调整糖尿病管理，改善贫血状况，并寻找乳酸升高的确切原因。

Coherence: 5, Factual consistency: 5, Evidence of statement: 4, Professionalism: 4 (Qwen)

Considering the complex medical history of this 78-year-old female patient, which includes hypertension, coronary heart disease, diabetes mellitus, hyperlipidemia, and glaucoma, as well as her current symptoms of shortness of breath and fatigue, there is reason to suspect multiple potential health issues. Firstly, shortness of breath and fatigue are common symptoms of heart failure, particularly in patients with a history of coronary heart disease. Heart failure can lead to a reduction in the heart's pumping capacity, resulting in impaired blood return and subsequent pulmonary congestion and hypoxia, which manifest as shortness of breath. Additionally, inadequate cardiac output can cause poor perfusion of the body's organs, leading to fatigue. Moreover, although the patient's hemoglobin and hematocrit are at the lower end of normal, they may still be associated with the chronic anemia often seen in heart failure. Secondly, diabetes mellitus and hyperlipidemia are risk factors for cardiovascular disease and may accelerate the process of atherosclerosis, further exacerbating the conditions of coronary heart disease and heart failure. These metabolic disorders may also impact renal function, leading to fluid and sodium retention, which can increase the cardiac workload. Furthermore, although glaucoma primarily affects the eyes, certain medications used in its treatment (such as beta-blockers) may have adverse effects on the cardiovascular system, exacerbating the symptoms of heart failure. In summary, the most likely diagnosis for this patient is heart failure, particularly in light of her history of coronary heart disease and the current symptoms. However, other potentialities cannot be excluded, such as pulmonary infection or acute exacerbation of chronic obstructive pulmonary disease, which may require further investigations (such as an electrocardiogram, chest X-ray, or echocardiogram) for confirmation. Additionally, for a diabetic patient, it is necessary to be vigilant for complications such as diabetic ketoacidosis; although the patient's lactate level is not significantly elevated, close monitoring of her blood glucose control is still essential. 考虑到这位 78 岁女性患者的复杂病史，包括高血压、冠心病、糖尿病、高脂血症和青光眼，以及她目前出现的憋气和乏力症状，我们有理由怀疑她可能存在多种潜在的健康问题。首先，憋气和乏力是心力衰竭的常见症状，特别是在有冠心病史的患者中。心力衰竭可能导致心脏泵血功能减弱，进而引起血液回流受阻，导致肺部淤血和缺氧，从而出现憋气。同时，心脏泵血功能不足也会导致全身器官供血不足，引发乏力。此外，患者血常规中的血红蛋白和红细胞压积虽然接近正常下限，但仍可能与心力衰竭引起的慢性贫血有关。其次，糖尿病和高脂血症都是心血管疾病的危险因素，它们可能加速动脉粥样硬化的进程，进一步加重冠心病和心力衰竭的病情。同时，这些代谢性疾病也可能影响肾脏功能，导致水钠潴留，进一步增加心脏负担。再者，青光眼虽主要影响眼部，但其治疗过程中使用的某些药物（如 受体阻滞剂）可能对心血管系统产生负面影响，加剧心力衰竭的症状。综合考虑，该患者最可能的诊断是心力衰竭，特别是考虑到她的冠心病史和当前的症状。然而，也不能排除其他潜在的可能性，如肺部感染或慢性阻塞性肺病急性加重等，这可能需要进一步的检查（如心电图、胸部 X 线或超声心动图）来确认。此外，对于糖尿病患者来说，还需要警惕酮症酸中毒等并发症的可能性，尽管该患者的乳酸水平并未显著升高，但仍需密切关注其血糖控制情况。

Coherence: 4, Factual consistency: 4, Evidence of statement: 4, Professionalism: 3 (Spark 4)

Table A7: Examples of AI-generated answers. Original Chinese texts are highlighted in red.

Question

A 78-year-old male patient with no history of smoking or alcohol use presents with chest pain, palpitations, nausea, fatigue, and shortness of breath upon exertion. The myocardial enzyme profile indicates a creatine kinase level of 79U/L and a troponin T level of 0.01ng/ml. What is the most likely diagnosis based on the patient's symptoms and laboratory findings? 一名 78 岁男性患者，无吸烟和饮酒史，主要表现为胸痛、心悸、恶心、乏力和活动后气短，心肌酶谱显示肌酸激酶为 79U/L，肌钙蛋白 T 为 0.01ng/ml。根据患者的症状和实验室检查结果，最可能的诊断是什么？

Answers

Based on the patient's symptoms and laboratory examination results, the most likely diagnosis is coronary heart disease (or stable angina). The patient is an elderly male without a history of smoking or alcohol consumption; however, he exhibits symptoms of chest pain, palpitations, nausea, fatigue, and shortness of breath upon exertion, which are common manifestations of coronary heart disease. The myocardial enzyme profile reveals that both creatine kinase and troponin T are within the normal range, suggesting that the patient has not experienced an acute myocardial infarction, but this does not rule out the possibility of coronary heart disease. Therefore, considering the patient's clinical presentation and laboratory findings, the most probable diagnosis is coronary heart disease (or stable angina). 根据患者的症状和实验室检查结果，最可能的诊断是冠心病（或稳定性心绞痛）。患者老年男性，虽无吸烟和饮酒史，但表现为胸痛、心悸、恶心、乏力和活动后气短，这些都是冠心病的常见症状。心肌酶谱显示肌酸激酶和肌钙蛋白 T 均处于正常范围，这可能表明患者未发生急性心肌梗死，但并不能排除冠心病的可能性。因此，综合患者的临床表现和实验室检查结果，最可能的诊断为冠心病（或稳定性心绞痛）。
Coherence: 4, Factual consistency: 3, Evidence of statement: 4, Professionalism: 4 (ERNIE 4)

According to the patient's symptoms and laboratory test results, the most probable diagnosis is stable coronary heart disease or mild myocardial ischemia. The patient presents with symptoms of chest pain, palpitations, nausea, fatigue, and shortness of breath upon exertion, which may be associated with cardiac disease. The myocardial enzyme profile shows a creatine kinase (CK) level of 79U/L, which is slightly elevated above the normal range (usually up to 55-170U/L), while the troponin T (cTnT) level is 0.01ng/ml, which is within the normal range (typically less than 0.01-0.03ng/ml). These indicators suggest that the extent of myocardial injury is mild and there is no obvious myocardial infarction. Therefore, given the patient's age and symptoms, stable coronary heart disease or mild myocardial ischemia is the most probable diagnosis. However, a definitive diagnosis requires the integration of further examination results, such as an electrocardiogram, echocardiography, and clinical assessment. 根据患者的症状和实验室检查结果，最可能的诊断是稳定性冠心病或轻微的心肌缺血。患者表现为胸痛、心悸、恶心、乏力和活动后气短，这些症状可能与心脏疾病有关。心肌酶谱显示肌酸激酶（CK）为 79U/L 略高于正常范围（通常上限为 55-170U/L），而肌钙蛋白 T（cTnT）为 0.01ng/ml，处于正常范围内（通常小于 0.01-0.03ng/ml）。这些指标表明心肌损伤的程度较轻，没有明显的心肌梗死。因此，考虑到患者的年龄和症状，稳定性冠心病或轻微的心肌缺血是最可能的诊断。然而，最终诊断需要结合心电图、心脏超声等进一步检查结果以及临床评估。
Coherence: 4, Factual consistency: 4, Evidence of statement: 4, Professionalism: 3 (ChatGLM 4)

Given the current symptoms presented (chest pain, palpitations, nausea, fatigue, shortness of breath after exertion) and the normal results of the myocardial enzyme profile, with creatine kinase (typically 50 - 310 U/L in males) and troponin T (typically less than 0.03ng/ml) within normal limits, a definitive diagnosis of myocardial infarction or other severe myocardial injury cannot be established. The most probable diagnosis should include consideration of other cardiovascular conditions, such as stable angina pectoris, arrhythmias, or heart failure. Additionally, it is possible that similar symptoms are caused by other systemic diseases, such as respiratory conditions (e.g., chronic obstructive pulmonary disease, although the patient's lack of a smoking history reduces the likelihood) or gastrointestinal diseases (e.g., gastroesophageal reflux disease causing chest pain, nausea, and other discomfort). Further relevant examinations, such as an electrocardiogram, echocardiography, ambulatory electrocardiogram monitoring, chest X-ray, or CT scan, are necessary to clarify the diagnosis. 仅根据目前提供的症状（胸痛、心悸、恶心、乏力、活动后气短）以及心肌酶谱中肌酸激酶（正常范围一般为男性 50 - 310 U/L）和肌钙蛋白 T（正常范围一般小于 0.03ng/ml）结果均正常，不能确诊为心肌梗死等严重心肌损伤疾病。最可能的诊断需要考虑其他心血管疾病，如稳定型心绞痛、心律失常或者心脏功能不全；也有可能是其他系统疾病引起类似症状，例如呼吸系统疾病（如慢性阻塞性肺疾病，不过患者无吸烟史相对可能性低一些）、消化系统疾病（如胃食管反流病引起的胸痛、恶心等不适）等，需要进一步完善相关检查如心电图、心脏超声、动态心电图监测、胸部 X 线或 CT 等来明确诊断。
Coherence: 4, Factual consistency: 4, Evidence of statement: 4, Professionalism: 4 (Doubao)

In response to the symptoms described (chest pain, palpitations, nausea, fatigue, and shortness of breath after exertion), along with normal creatine kinase and troponin T levels in the myocardial enzyme profile, which are typically within the ranges of 50 - 310 U/L for males and less than 0.03ng/ml respectively, a diagnosis of myocardial infarction or other severe myocardial injury cannot be confirmed. The most probable diagnoses should consider other cardiovascular conditions, such as stable angina pectoris, arrhythmias, or heart failure. Additionally, it is possible that similar symptoms are caused by other systemic diseases, such as respiratory conditions (e.g., chronic obstructive pulmonary disease, although the patient's lack of a smoking history reduces the likelihood) or gastrointestinal diseases (e.g., gastroesophageal reflux disease causing chest pain, nausea, and other discomfort). Further relevant examinations, such as an electrocardiogram, echocardiography, ambulatory electrocardiogram monitoring, chest X-ray, or CT scan, are necessary to clarify the diagnosis. 根据患者的年龄、症状以及实验室检查结果，最可能的诊断是冠状动脉粥样硬化性心脏病（冠心病）。患者虽然没有吸烟和饮酒史，但胸痛、心悸、恶心、乏力和活动后气短都是冠心病的典型症状。心肌酶谱中的肌酸激酶和肌钙蛋白 T 水平虽然未显著升高，但这并不排除冠心病的可能性，特别是在高龄患者中。建议进一步进行心电图、心脏超声等相关检查以明确诊断。
Coherence: 3, Factual consistency: 3, Evidence of statement: 3, Professionalism: 3 (Hunyuan)

Non-ST Elevation Acute Coronary Syndrome (NSTEMI). Explanation: * The patient's symptoms, including chest pain, palpitations, nausea, fatigue, and shortness of breath after exertion, suggest the presence of a cardiac-related condition. * The myocardial enzyme profile reveals a creatine kinase (CK) level of 79U/L and a troponin T (cTnT) level of 0.01ng/ml. The CK level is slightly elevated above the normal range, while the cTnT level is within the normal range. This suggests possible myocardial damage, albeit atypical. * Based on the symptoms and laboratory test results, ST-segment elevation acute coronary syndrome (STEMI) can be excluded, and the possibility of non-ST-segment elevation acute coronary syndrome (NSTEMI) is considered. * NSTEMI is an acute coronary syndrome characterized by chest pain or discomfort and evidence of myocardial damage without ST-segment elevation on the electrocardiogram. 非 ST 段抬高型急性冠状动脉综合征（Non-ST Elevation Acute Coronary Syndrome，NSTEMI）解释：* 患者的症状包括胸痛、心悸、恶心、乏力和活动后气短，提示可能存在心脏相关疾病。* 心肌酶谱结果显示肌酸激酶（CK）为 79U/L，肌钙蛋白 T（cTnT）为 0.01ng/ml。CK 略高于正常值，而 cTnT 在正常范围内。这表明可能存在心肌损害，但不典型。* 根据症状和实验室检查结果，排除 ST 段抬高型急性冠状动脉综合征（STEMI），考虑非 ST 段抬高型急性冠状动脉综合征（NSTEMI）的可能性。* NSTEMI 是一种急性冠状动脉综合征，caracterized by chest pain or discomfort and evidence of myocardial damage without ST-segment elevation on the electrocardiogram.[1]
Coherence: 4, Factual consistency: 4, Evidence of statement: 4, Professionalism: 4 (Llama 3 70B)

Based on the patient's symptoms (chest pain, palpitations, nausea, fatigue, and shortness of breath after exertion) and laboratory test results (creatine kinase 79U/L, troponin T 0.01ng/ml), the most probable diagnosis is myocardial infarction or myocardial damage. 基于患者的症状（胸痛、心悸、恶心、乏力和活动后气短）和实验室检查结果（肌酸激酶为 79U/L、肌钙蛋白 T 为 0.01ng/ml），最可能的诊断是心肌梗死或心肌损害。
Coherence: 3, Factual consistency: 3, Evidence of statement: 3, Professionalism: 3 (Mistral 7B)

Given the patient's presentation (chest pain, palpitations, nausea, fatigue, and shortness of breath after exertion) and the results of the myocardial enzyme profile (creatine kinase 79U/L, troponin T 0.01ng/ml, with a mildly elevated troponin T being a key finding), the most likely diagnosis is acute coronary syndrome (including stable angina or non-ST segment elevation myocardial infarction). It is important to note that a definitive diagnosis requires a detailed assessment by a clinical physician, the results of an electrocardiogram, and other relevant tests. 根据患者的表现（胸痛、心悸、恶心、乏力和活动后气短）以及心肌酶谱的结果（肌酸激酶为 79U/L，肌钙蛋白 T 为 0.01ng/ml，其中肌钙蛋白 T 轻度升高较为关键），最可能的诊断是急性冠状动脉综合征（包括心绞痛或非 ST 段抬高型心肌梗死）。需要注意的是，最终确诊需要结合临床医生的详细评估、心电图检查结果以及其他相关检查。
Coherence: 3, Factual consistency: 3, Evidence of statement: 3, Professionalism: 4 (Qwen)

Acute Coronary Syndrome. Explanation: The 78-year-old male patient presents with symptoms of chest pain, palpitations, nausea, fatigue, and shortness of breath after exertion, which are all typical manifestations of acute coronary syndrome (ACS). The myocardial enzyme profile shows a creatine kinase level of 79U/L and a troponin T level of 0.01ng/ml, which are consistent with the laboratory findings of ACS. Although this patient has no history of smoking or alcohol use, his age, gender, and other risk factors (such as hypertension, diabetes, high cholesterol, etc.) may still increase his susceptibility to developing acute coronary syndrome. Therefore, based on his symptoms and laboratory test results, the most probable diagnosis is acute coronary syndrome. 急性冠状动脉综合征解释：这名 78 岁的男性患者表现出胸痛、心悸、恶心、乏力和活动后气短，这些都是急性冠状动脉综合征（ACS）的典型症状。心肌酶谱显示肌酸激酶为 79U/L，肌钙蛋白 T 为 0.01ng/ml，这也与急性冠状动脉综合征的实验室检查结果相符。虽然这位患者没有吸烟和饮酒史，但年龄、性别和其他风险因素（如高血压、糖尿病、高胆固醇等）仍然可能增加他患上急性冠状动脉综合征的风险。因此，根据他的症状和实验室检查结果，最可能的诊断是急性冠状动脉综合征。
Coherence: 4, Factual consistency: 4, Evidence of statement: 4, Professionalism: 4 (Spark 4)

[1]The machine-generated response includes texts in English.

Fig. A2: (a) Distribution of the number of words in relation to the evaluation across four metrics for question generation. (b) Distribution of the number of words in relation to the evaluation across four metrics for question answering. (c) Distribution of the proportion of novel words of the AI-generated content over the source input in relation to the evaluation across four metrics for question generation. (d) Distribution of the proportion of novel words of the AI-generated content over the source input in relation to the evaluation across four metrics for question answering.

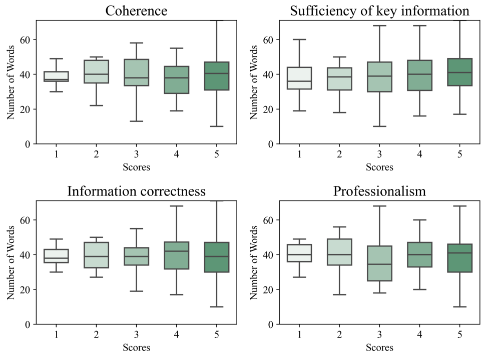

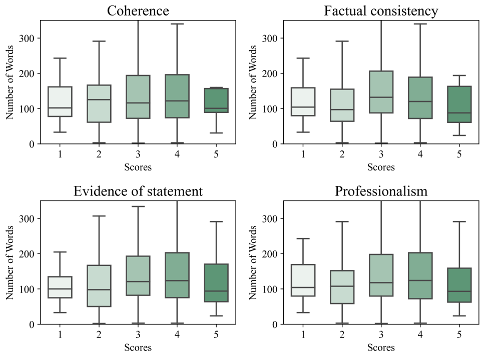

(a) (b)

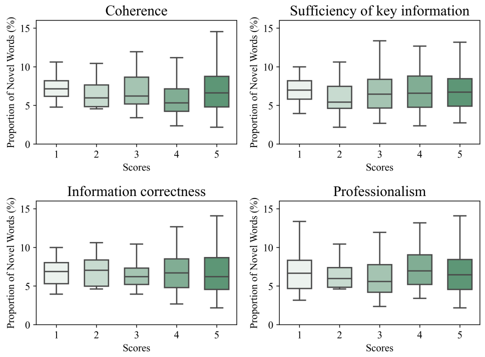

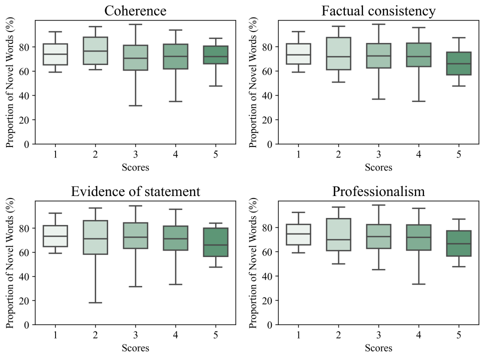

(c) (d)
